# Explainable Encoder–Prediction–Reconstruction Framework for the Prediction of Metasurface Absorption Spectra

**DOI:** 10.3390/nano14181497

**Published:** 2024-09-14

**Authors:** Yajie Ouyang, Yunhui Zeng, Xiaoxiang Liu

**Affiliations:** 1 School of Intelligent Systems Science and Engineering, Jinan University, Zhuhai 519070, China; oyyj2003@outlook.com (Y.O.); tlxx@jnu.edu.cn (X.L.); 2 Shenzhen International Graduate School, Tsinghua University, Shenzhen 518055, China

**Keywords:** metasurface, deep learning, explainability, absorption spectra

## Abstract

The correlation between metasurface structures and their corresponding absorption spectra is inherently complex due to intricate physical interactions. Additionally, the reliance on Maxwell’s equations for simulating these relationships leads to extensive computational demands, significantly hindering rapid development in this area. Numerous researchers have employed artificial intelligence (AI) models to predict absorption spectra. However, these models often act as black boxes. Despite training high-performance models, it remains challenging to verify if they are fitting to rational patterns or merely guessing outcomes. To address these challenges, we introduce the Explainable Encoder–Prediction–Reconstruction (EEPR) framework, which separates the prediction process into feature extraction and spectra generation, facilitating a deeper understanding of the physical relationships between metasurface structures and spectra and unveiling the model’s operations at the feature level. Our model achieves a 66.23% reduction in average Mean Square Error (MSE), with an MSE of 2.843 $\times \;10^{-4}$ compared to the average MSE of $8.421\times 10^{-4}$ for mainstream networks. Additionally, our model operates approximately 500,000 times faster than traditional simulations based on Maxwell’s equations, with a time of $3\times 10^{-3}$ seconds per sample, and demonstrates excellent generalization capabilities. By utilizing the EEPR framework, we achieve feature-level explainability and offer insights into the physical properties and their impact on metasurface structures, going beyond the pixel-level explanations provided by existing research. Additionally, we demonstrate the capability to adjust absorption by changing the metasurface at the feature level. These insights potentially empower designers to refine structures and enhance their trust in AI applications.

## 1. Introduction

The control of electromagnetic responses in optical structures, particularly nanophotonic structures, is a key focus of current research. Unlike conventional materials, whose properties like transmittance, refractive index, and absorption are dictated by their intrinsic characteristics, metamaterials derive their properties from artificially designed structures. This design flexibility allows for the creation of metamaterials with unique properties not found in nature. Metasurfaces, as two-dimensional variants of metamaterials, effectively modulate electromagnetic properties, demonstrating superior performance phase [1], polarization [2,3], and wavefront [4] control.

Due to their precise electromagnetic modulation capability, compactness, ease of integration, and versatility, metasurfaces are widely used in optical imaging [5,6], biomedicine [7,8], and communications [9,10,11]. Notably, the control of absorption properties is vital for applications such as photonic absorbers [12], enhanced generation of hot electrons [13], solar absorbers [14,15,16,17], and infrared (IR) stealth technology [18]. As the demand for advanced capabilities and high-performance nanophotonic devices grows, controlling the absorption spectra of metasurfaces is of great interest.

Designing metasurfaces requires comprehensive simulations that predominantly entail solving Maxwell’s equations, which are typically conducted using advanced simulation platforms such as Lumerical FDTD and CST STUDIO SUITE. These simulations are not only time-intensive but also demand substantial computational resources, thus posing significant limitations on the pace of research and innovation in the field of metasurfaces. Therefore, it is crucial to predict the electromagnetic responses of metasurface structures quickly and accurately.

In recent years, artificial intelligence methods have been successfully applied in many fields, such as disaster risk prediction [19], natural language processing [20,21], production scheduling [22,23], and medical diagnosis [24,25,26,27,28]. The field of nanophotonics, which involves the manipulation of light at the nanoscale, has similarly benefited from these advancements. Numerous studies have leveraged deep neural networks (DNNs) to uncover the complex relationships between the electromagnetic responses of nanostructures and their feature spaces [29,30,31]. With the increasing complexity of metasurface structures designed for higher performance, traditional methods that rely on discrete parameter vectors are becoming inadequate [18,32]. Consequently, employing image coding techniques, such as using three-channel images, becomes essential to effectively represent the freeform shapes and heterogeneous material properties of each part of the metasurface [33,34,35]. However, as the parameter space expands, models become increasingly complex, making it more challenging to understand their inner workings. Existing research primarily focuses on using neural networks to fit structural parameters to electromagnetic responses, often resulting in black box models that are difficult to interpret. This lack of transparency makes it challenging for designers to determine whether the models are accurately extracting key features or merely fitting arbitrary patterns. Consequently, designers may be hesitant to rely on predictions from models that cannot be easily explained, limiting the broader application of DNNs in metasurface design.

As a result, there remains a need for greater emphasis on the aspect of explainability in current research. Borui et al. [36] utilized the equivalent transmission line theory to predict impedance by physically constrained deep learning algorithms and an improved genetic algorithm (GA). However, they only used the explicit equivalent-circuit-intervened model as a guide for the implicit deep learning method and did not focus on the explainability of the model. This increases the model design’s complexity, requiring an in-depth understanding of equivalent circuits and neural networks. Yeung et al. [37] verified that the Explainable Artificial Intelligence (XAI) method could extract the patterns and principles encoded in machine learning (ML) models, thus gaining valuable insights into the behavior of nanophotonic structures. Nevertheless, they simply applied the Shapley Additive Explanation (SHAP) [38] method to interpret the prediction results of the trained model without considering the network architecture, thereby failing to gain a deeper understanding of the model’s working principles. Moreover, their interpretations were limited to the pixel level, which does not align with human thinking habits, making it difficult to gain designers’ understanding and trust.

Additionally, unlike simpler tasks such as image classification or object detection, explainability in predicting electromagnetic responses presents greater challenges. In image categorization tasks, humans can easily distinguish objects directly based on the image, making it simpler to understand how the model works. It is only necessary to identify a set of pixels that significantly contribute to the category. If these pixels are concentrated on a particular object, humans can determine whether the model extracts the correct features to distinguish between different objects. However, predicting the absorption spectrum from a metasurface structure is far more complex. Metasurfaces often operate at the nanoscale, where small variations in design parameters can lead to significant changes in electromagnetic responses. Even professionals cannot derive a rough prediction of the absorption spectrum just by examining a metasurface structure. Therefore, calculating the SHAP value [39] for each pixel and identifying the model’s region of interest does not clarify why these pixels enable specific predictions. Additionally, if all of these pixels are concentrated in the edge regions of the image, it remains uncertain whether the model has extracted the key features for accurate prediction or understood the physical relationship between the structure and the electromagnetic response. Convolutional Neural Networks often extract edge information in image-related tasks, raising the possibility that the network is merely memorizing image features and their corresponding spectra to establish one-to-one correspondences. What is truly needed is an understanding of the features extracted by the model—whether they reflect the structure’s shape or material properties and which features are most influential in making predictions. For instance, we need to understand how the material and thickness of a resonator impact the prediction results and determine which factor is more significant, rather than just knowing that a specific set of pixels is crucial for the predictions. Hence, explainability in predicting electromagnetic responses is more challenging and cannot be achieved by simply applying any XAI method. There is an urgent need for an effective method that achieves feature-level (physically meaningful) explainability while maintaining high accuracy and generalization performance.

Based on this need, we propose a new framework called the Explainable Encoder–Prediction–Reconstruction (EEPR) framework, designed for predicting metasurface absorption spectra. This framework aims to balance high performance with feature-level explainability, thereby achieving both accuracy and a deeper understanding of the underlying physical features. We use an Encoder–Decoder architecture to extract the features of the structure and encode them into embedding vectors before performing spectral prediction. Further, pre-training and fine-tuning are used to ensure the model extracts features before prediction. A reconstruction network is trained to reconstruct the embedding vectors into metasurface structures and combined with the SHAP method to improve the explainability of the model. The main contributions of this paper are summarized as follows:

1. **Development of an Explainable Framework**: We introduce a novel EEPR framework for predicting metasurface absorption spectra, balancing high performance with explainability. The framework clarifies the importance and significance of the extracted features by integrating SHAP methods and a reconstruction network while enhancing the ability to extract effective features using an Encoder–Decoder architecture and pre-training strategies. By examining the impact of the extracted features on the metasurface, we demonstrate that these features reflect physical significance.
2. **Enhancement of Prediction Efficiency**: Our proposed model significantly reduces simulation time while maintaining high accuracy in predicting absorption spectra. This improvement offers a practical alternative to traditional simulation software, enhancing efficiency in metasurface design. Additionally, it can be applied in fields requiring rapid responses, such as interactions between agents and environments in reinforcement learning.
3. **Scalable and User-Friendly Approach**: The EEPR framework is scalable and can be generalized to predict the electromagnetic responses of various nanophotonic devices. It presents explainability results in an intuitive manner and is easy to implement, enabling designers to use the framework without extensive artificial intelligence (AI) expertise.

## 2. Methods

### 2.1. Proposed Framework

**Figure 1:**
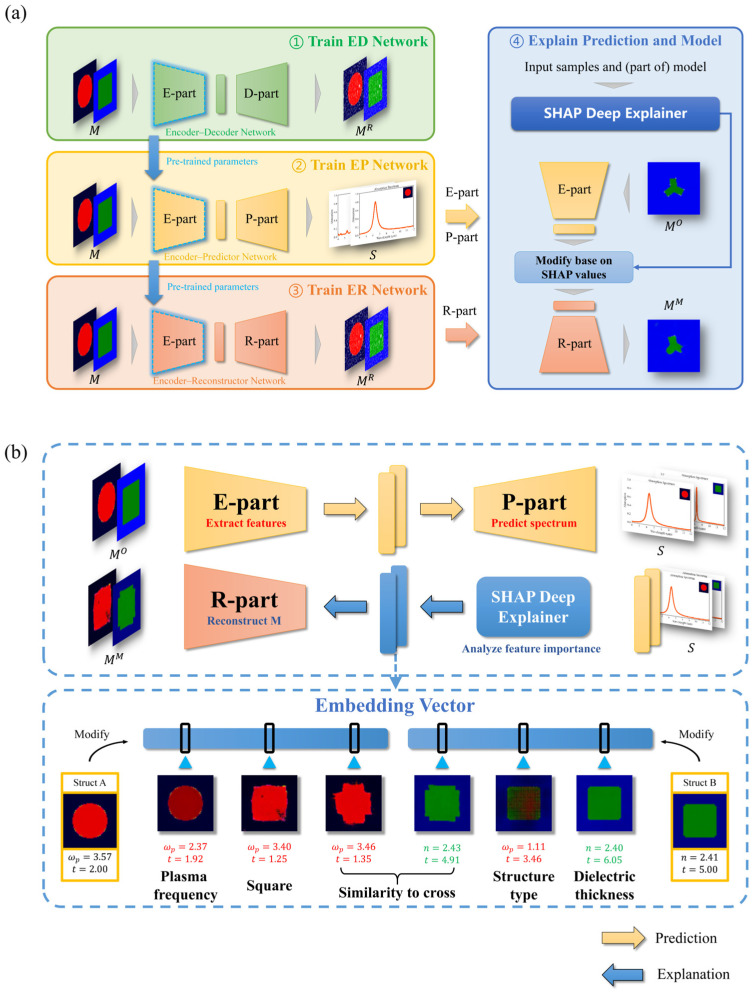
The EEPR framework. (**a**) Flowchart of the EEPR framework. (**b**) Overview of explanation at the feature level, where M is the metasurface structure as the input of the three networks, $M^{R}$ is the metasurface structure obtained by reconstruction with the ED Network or the ER Network, S is the corresponding absorption spectrum predicted by the EP Network, and $M^{M}$ is the modified metasurface structure obtained by adjusting the embedding vectors based on the original metasurface structure (structure for analysis) $M^{O}$.

Our proposed EEPR framework for metasurface absorption spectra prediction consists of the Encoder–Decoder Network (ED Network, including E-part and D-part), the Encoder–Predictor Network (EP Network, including the E-part and P-part), and the Encoder–Reconstructor Network (ER Network, including the E-part and R-part). The workflow of the EEPR framework is illustrated in Figure 1a, with the mathematical principles detailed in Section 2.3. The overall process of the framework is as follows: (1) Train the ED Network using the metasurface structures in the dataset. (2) Train the EP Network using the metasurface structures and the corresponding absorption spectra in the dataset. The E-part of the EP Network uses the pre-trained parameters of the E-part in the ED Network as the initialization parameters, and the E-part and P-part of the EP Network are trained at different learning rates. (3) Train the ER Network using the metasurface structures in the dataset. The ER Network’s E-part uses the E-part from the EP Network as initialization parameters and freezes them. In other words, the E-part of the ER Network and the EP Network are kept consistent, and only the R-part is trained to ensure that the same metasurface structure receives the same embedding vectors in the EP Network and the ER Network, which makes it possible for the R-part to reconstruct the embedding vectors into the corresponding metasurface structure. (4) Explain Prediction and Model: in addition to using the SHAP Deep Explainer to interpret the EP Network directly, we can also extract the embedding vector outputs from the E-part in the EP Network and input them into the SHAP Deep Explainer [38,40,41], as detailed in Section 3.1, together with the P-part to obtain the SHAP values of each dimension of the embedding vectors. Modifying the embedding vectors according to the SHAP value and inputting them into the R-part of the ER Network to obtain the modified metasurface structure can further open the black box of the model. By modifying one or more dimensions of the embedding vectors with larger SHAP values, it is possible to visualize the meaning of the features extracted by the model and understand the principle of the network making predictions. This is described in detail in Figure 1b. Further, combined with specific needs (e.g., increasing or decreasing the absorption value), modifying the feature dimensions in the embedding vectors according to the SHAP value is positive or negative, enabling the generation of new metasurface structures by changing the existing ones at the feature level.

An overview of the explanation at the feature level is shown in Figure 1b. The E-part extracts the features to obtain the embedding vector (where each dimension represents a feature), and then the absorption spectrum is predicted by the P-part. Using the SHAP Deep Explainer, we can identify the model’s regions of interest and determine the importance of each feature within the embedding vector. After modifying a feature in the embedding vector, the R-part reconstructs the metasurface structure, thereby elucidating the feature’s significance and its impact on the structure. Combined with SHAP Deep Explainer and our unique network architecture, a feature-level interpretation of the model is made. In the figure, Struct A and Struct B are different types of metasurface structures randomly selected from the dataset. In Struct A, specific features corresponding to the plasma frequency are extracted, exhibiting insensitivity to other data, whereas other features emphasize the resonator shape, delineating its resemblance to either a square or a cross. In Struct B, specific features extract the thickness of the dielectric layer, demonstrating insensitivity to other data, while other features focus on the structural type, with alterations in their dimensions leading to modifications in structural type. Moreover, the same feature dimension in the embedding vector can control the degree of similarity between the shape of the resonator and the cross shape in Struct A and Struct B. This indicates that the feature can extract the shape of the resonator for different structural types. This part will be discussed in detail in Section 3.3.

### 2.2. Network Architecture

The task of absorption spectra prediction is challenging, requiring precise prediction of both the spectral shape and absorption values across multiple wavelengths. Small changes in the shape or material properties of the metasurfaces can significantly impact the absorption spectra, so a high-performance and efficient model is needed to identify the different shapes of structures and material properties to predict the absorption spectra correctly.

**Figure 2:**
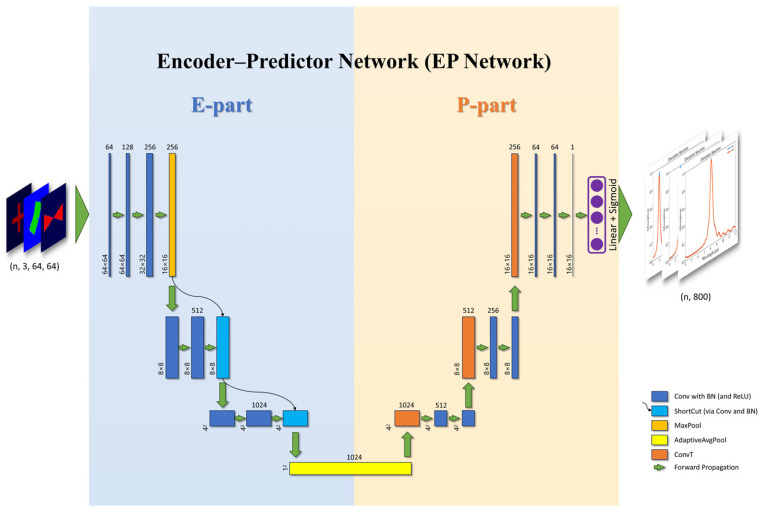
Architecture of the EP Network. Conv: convolutional layer; BN: batch normalization layer; MaxPool: max pooling layer; AdaptiveAvgPool: adaptive average pooling layer; and ConvT: transposed convolutional layer.

However, existing research works on predicting metasurface absorption spectra use models such as fully connected networks [18], traditional Convolutional Neural Networks (CNNs) [13], and ResNet [16,42], which directly map the metasurface structure into absorption spectra, resulting in limited learning capability and explainability. The correlation between metasurface structures and their corresponding electromagnetic response is inherently complex, and direct mapping often fails to yield optimal predictive performance. An alternative method involves extracting critical features into a latent space, and subsequently predicting the electromagnetic responses based on these features. To enable the network to extract the critical features in the structure and enhance the explainability of the network, our forward prediction model (EP Network) employs an Encoder–Decoder architecture, which explicitly separates the prediction process into feature extraction and spectral generation, as shown in Figure 2, where each block represents a multi-channel feature map after passing through the corresponding network layer, with the size of the feature map labeled at the bottom left, the number of channels labeled at the top, and the green arrows represent the direction of forward propagation of the network. The input to the prediction network is a batch of pre-processed (to allow the model to learn better, we normalize the RGB-encoded metasurface data with a mean of 0 and standard deviation of 0.5) RGB images in the shape of 3 × 64 × 64, where 3 is the number of channels, and the rest are the width and height of the image. After a series of convolution, pooling, and deconvolution operations, the output is a batch of 800-dimensional vectors, i.e., absorption spectra, through a fully connected layer and a sigmoid activation function.

The network extracts features in the metasurface structure useful for spectral prediction in the E-part and makes predictions based on the extracted features in the P-part. In the E-part, we employ the ResNet architecture [37], which addresses the challenges of training deeper networks by mitigating vanishing or exploding gradients, thus facilitating more effective feature extraction and enhancing the network’s learning capabilities. In the P-part, we use an inverse convolutional layer to achieve upsampling and make the P-part as symmetric as possible with the E-part. This Encoder–Decoder architecture is designed to improve prediction accuracy and explainability by leveraging explicit feature extraction.

To enhance the explainability and training efficiency, the number of parameters of the model should be manageable. Therefore, in the middle part of the network, we use an adaptive average pooling layer to compress the multi-channel feature maps to a size of 1 × 1, resulting in an embedding vector that serves as both the output of the E-part and the input of the P-part. This embedding vector is smaller in dimension than the multi-channel feature map, making it easier to analyze and suitable for downstream tasks such as clustering, metasurface structure reconstruction, and feature analysis.

Additionally, to ensure that the model extracts features before prediction, we initially trained the ED Network to extract critical features and encode high-dimensional metasurface structures into low-dimensional embedding vectors. Subsequently, the pre-trained parameters of the E-part in the ED Network were used as initialization parameters for the E-part of the EP Network. Since the encoding method of the ED Network is a lossy compression that only encodes the original image and is not specifically tailored for absorption spectra prediction, it may lose critical information. Therefore, after inheriting the ED Network’s pre-trained parameters, the EP Network’s E-part continues to be trained to adapt to the absorption spectra prediction task. Therefore, after transferring the pre-trained parameters from the ED Network, the E-part and P-part of the EP Network are further trained at different learning rates to adapt to the absorption spectra prediction task.

In the training process of the prediction network, we use the Spectral Overlap Coefficient (SOC) as the loss function, a metric proposed in our previous work [43]. The formula for the SOC is as follows:

(1) $$SOC=1-\frac{\sum_{i}^{n}min(S_{i}^{P},\;S_{i}^{G})}{\sum_{i}^{n}max(S_{i}^{P},\;S_{i}^{G})}$$

where $S_{i}^{P}$ and $S_{i}^{G}$ represent the absorption value at the ith wavelength in the predicted and ground truth spectra, respectively. Here, we use 800 points to describe the absorption spectrum from 4 to 12 μm, i.e., i takes the value from 1 to 800. The SOC measures the degree of overlap between the two spectra and precisely quantifies their similarity, which ranges from 0 to 1, with closer to 0 indicating that the two spectra are more similar and closer to 1 indicating that they are less similar. The SOC, providing a more nuanced assessment of spectral similarity, has proved more effective for spectra prediction tasks than the traditional MSE (Mean Square Error) loss function [43]. Detailed advantages of using the SOC as a loss function are provided in the Supplementary Materials (Table S1).

Overall, the EP Network has two main features: (1) it employs an Encoder–Decoder architecture; (2) it uses an adaptive average pooling layer to compress the size of the multi-channel feature maps in the middle of the network to 1 × 1, resulting in an embedding vector. These features enhance prediction accuracy and explainability and provide a foundation for integrating the ED Network and the ER Network. Additionally, using the SOC as the loss function, rather than the MSE, improves prediction accuracy to some extent. The architectures of the ED Network (using the MSE as the loss function) and the ER Network (using the MSE as the loss function) are detailed in the Supplementary Materials (Figures S1 and S2).

### 2.3. Mathematical Principles of the EEPR Framework

Our proposed EEPR framework uses the SHAP method. SHAP is an XAI method, a technique for opening the black box of a model by assigning each feature an importance value for a particular prediction, known as the SHAP value, which is used as a measure of the feature’s contribution to the prediction. The greater the SHAP value, the greater the influence of the feature on the prediction result. Compared to other XAI methods, SHAP not only highlights the regions of interest within a model but also differentiates the impacts of these regions on the predictions, whether positive or negative. If the SHAP value is positive, the feature positively influences the prediction; if the SHAP value is negative, it inhibits the prediction.

For the original prediction model (a complex model f, e.g., a deep network) to be explained, it is common to use the simpler explanation model g, which uses simplified inputs $x^{\prime }$ that map to the original inputs through a mapping function $x=h_{x}(x^{\prime })$ (for the current input x), $g(z^{\prime })\approx f(h_{x}(z^{\prime }))$ whenever $z^{\prime }=x^{\prime }$. $h_{x}$ maps 1 or 0 to the original input space, where 1 indicates the input is included in the model, and 0 indicates exclusion from the model. The classic Shapley value equations are as follows:

(2) $$\phi_{i}(f,x)=\sum_{z^{\prime }\subseteq x^{\prime }}\;\frac{\left|z^{\prime }\right|!\left(M-\left|z^{\prime }\right|-1\right)!}{M!}\left[f_{x}\left(z^{\prime }\right)-f_{x}\left(z^{\prime }\setminus i\right)\right]$$

where the values $\phi_{i}\in R$ are known as Shapley values, $f_{x}\left(z^{\prime }\right)=f\left(h_{x}\left(z^{\prime }\right)\right)$, $z^{\prime }\in \{0,1{\}}^{M}$, M is the number of simplified input features, $z^{\prime }\setminus i$ denote setting $z_{i}^{\prime }=0$, $\left|z^{\prime }\right|$ is the number of non-zero entries in $z^{\prime }$, and $z^{\prime }\subseteq x^{\prime }$ represents all $z^{\prime }$ vectors where the non-zero entries are a subset of the non-zero entries in $z^{\prime }$.

SHAP values are the Shapley values of a conditional expectation function of the original model, where, $f_{x}\left(z^{\prime }\right)=f\left(h_{x}\left(z^{\prime }\right)\right)=E\left[f(z)|z_{N}\right]$, and N is the set of non-zero indexes in $z^{\prime }$. It is worth noting that the SHAP additive feature attribution method is based on a linear function of binary values as follows:

(3) $$g\left(z^{\prime }\right)=\phi_{0}+\sum_{i=1}^{M}\;\phi_{i}z_{i}^{\prime }$$

where $\phi_{0}$ is the baseline value (theoretically interpreted as the prediction when all features are missing). In practice, $\phi_{0}$ is typically implemented as the average prediction over the dataset:

(4) $$\phi_{0}=\frac{1}{\left|D\right|}\sum_{x\subset D}\;g(x)$$

where D is the dataset and g(x) is the model’s prediction for instance x. Additive feature attribution is a key property of SHAP, meaning that the model’s output can be decomposed into a linear combination of the contributions from each feature. This property enables SHAP to provide consistent and intuitive explanations. In simple terms, this means that the sum of all Shapley values and the average predicted value is equal to the prediction, as detailed in Figure S3.

For the task of predicting metasurface absorption spectra, we typically focus on analyzing the peaks in the spectrum. To perform feature-level explanation, we first input the P-part of the EP Network and the embedding vectors of the samples into the SHAP Deep Explainer to obtain the SHAP values for each dimension (feature) in the embedding vectors. This process allows us to assess the influence of each dimension on the prediction results. Subsequently, we modify a specific dimension i (referred to as Feature i) in the embedding vector by multiplying it by the feature factor F. The resulting modified embedding vector is as follows:

(5) $$V_{i}^{M}=V_{i}^{O}\times F$$

where $V_{i}^{M}$ denotes the ith dimension of the modified embedding vector and $V_{i}^{O}$ denotes the ith dimension of the original embedding vector. The modified embedding vector is then input into the R-part of the ER Network to obtain the reconstructed metasurface structure. Modifying the feature factor F allows us to visualize the impact of this dimension on the metasurface structure. A schematic of this process is shown in Figure S4. In addition, the E-part of the EP Network and the metasurface structures are input into the SHAP Deep Explainer to obtain the SHAP values for each pixel in the metasurface structures. By simultaneously analyzing the heatmap of the absolute SHAP values and observing the effects of modifying feature factors on the metasurface structure, we can more accurately identify the features extracted from specific dimensions of the embedding vector.

To modify the existing metasurface at the feature level and generate new metasurface designs, we leverage the SHAP values as follows: A positive SHAP value at a specific wavelength indicates that the feature promotes absorption, while a negative value suggests it is inhibitory. Based on this property, we divide all feature dimensions into two groups—those with positive SHAP values and those with negative SHAP values—and then multiply them by the positive factor $F_{p}$ and negative factor $F_{n}$, respectively. Thus, for a particular feature dimension, its modified value is as follows:

(6) $$V_{i}^{M}=\left\{\begin{array}{c} V_{i}^{O}\times F_{p},\;V_{i}^{SHAP}>0\\ V_{i}^{O}\times F_{n},\;V_{i}^{SHAP}<0 \end{array}\right.$$

where $V^{O}$ is the original embedding vector and $V^{M}$ is the modified embedding vector. If $F_{p}>1,F_{n}<1$, then the absorption value at that wavelength rises; otherwise, it falls. The modified embedding vector is input into the R-part to obtain the reconstructed structure, and the absorption spectrum of the new structure can be obtained by inputting it into the EP Network for prediction. A schematic of this process is shown in Figure S5. In addition, the magnitude of the rise or fall of the absorption values can be controlled by adjusting the magnitude of the positive and negative factors.

## 3. Results and Discussion

### 3.1. Experimental Setup and Data Sources

We evaluated our model using an open-source dataset [33] comprising 18,768 samples after filtering. Approximately 90% of the samples were used for training, and the remaining 10% were reserved for testing. Details on model training are provided in the Supplementary Materials (Figures S6–S8).

In this dataset, the structures of the metasurfaces are represented by three-channel RGB images, comprising two types of absorbing metasurfaces: metal–insulator–metal (MIM) structures and hybrid dielectric structures. The R channel encodes the plasma frequency of the MIM structure’s resonator, the G channel encodes the refractive index of the hybrid dielectric structure’s resonator, and the B channel encodes the thickness of the dielectric layer for both types of structures. Moreover, all three channels can represent the shape of the resonator. For convenience, we refer to the R channel, G channel, and B channel as the plasma frequency channel ($\omega_{p}$ channel), the refractive index channel (n channel), and the dielectric thickness channel (t channel), respectively. Detailed information can be found in the Supplementary Materials (Figure S9).

Due to the high computational cost of SHAP values, we typically use Python packages [41] to approximate SHAP values in engineering practice. In this study, we employ the SHAP Deep Explainer to analyze the model. The experiments were conducted on a machine equipped with an Intel(R) Core(TM) i7-14700KF CPU and a single NVIDIA GeForce RTX 4090D GPU.

### 3.2. Forward Prediction Model Evaluation

To demonstrate the performance of our EP Network, we compared it with some mainstream models and the results are presented in Table 1. All models in the table were trained using the SOC as the loss function and achieved convergence after 500 epochs. The SOC loss of our proposed framework on the test set is 0.079, and the MSE loss is 2.843$\times 10^{-4}$. Our model outperforms all other models on the test set, as evaluated using both SOC and MSE metrics. The average MSE for other mainstream networks is $8.421\times 10^{-4}$, while the optimal MSE is $5.190\times 10^{-4}$. Furthermore, our network gains a distinct advantage from the pre-training with parameters from the ED Network, an approach not feasible for the other models listed due to architectural constraints. Leveraging the pre-trained parameters from the ED Network, our model achieves an MSE that is 66.23% lower than the average MSE of other mainstream networks and 45.22% lower than their optimal MSE. The parameter sizes and training times for each model are detailed in Table S2, and the hyperparameter conditions detailed in Table S3.

We observe that although UNet and ResNet exhibit lower loss on the training set compared to our model, their performance on the test set is less consistent, indicating that these models may rely more on memorizing input–output mappings rather than capturing the underlying physical relationships between metasurface structures and absorption spectra. In contrast, our model demonstrates better generalization, effectively mitigating overfitting and showing much lower performance discrepancy between the training and test sets.

**Figure 3:**
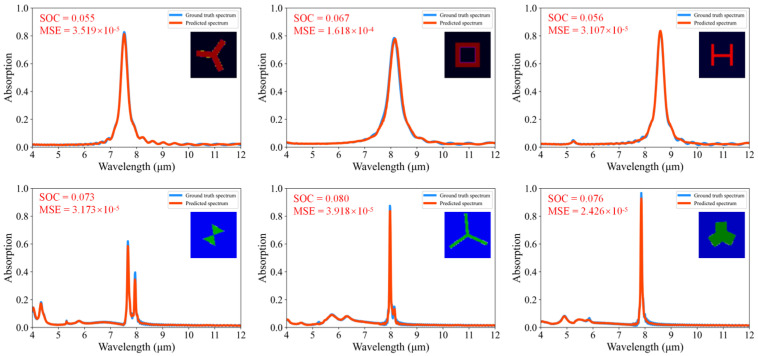
Comparison between predicted and ground truth spectra.

To visualize the model’s performance, we randomly selected six samples from the test set. Figure 3 compares the absorption spectra predicted by the model with the ground truth spectra. This comparison clearly illustrates that our model’s predictions are highly accurate, underscoring the effectiveness of our approach.

We tested each sample on the test set, and the time required to acquire the predicted absorption spectra was 5.757 s, averaging $3\times 10^{-3}$ seconds for one sample, whereas 25 min for one sample using the finite-difference time-domain (FDTD) simulation (about 5 min for the MIM structures and about 45 min for the hybrid dielectric structures). In comparison, the time consumed to obtain absorption spectra using our model is approximately 500,000 times faster than the FDTD simulation. Detailed information about the FDTD simulations can be found in the Supplementary Materials (Tables S4 and S5).

To validate the model’s generalization capability, we designed a case study in which we encoded metasurfaces significantly different from the samples in the dataset and input them into the model for prediction. By comparing our model with other mainstream networks, we demonstrate its superior generalization performance. Details of this case study can be found in the Supplementary Materials (Figure S10).

### 3.3. Explain Prediction and Model

First, we implemented the explanation at the pixel level using SHAP. Subsequently, we provided feature-level explanations using the EEPR framework. Based on this, we modified the metasurface structure at the feature level to control absorption. By comparing these results with pixel-level explanations, we demonstrate the advantages of our feature-level approach.

#### 3.3.1. Explanation at the Pixel Level

**Figure 4:**
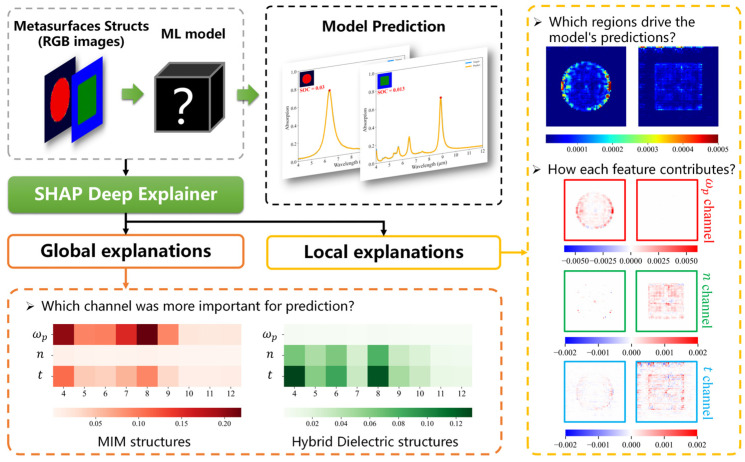
The pixel-level explanation process.

The pixel-level explanation process is shown in Figure 4. Initially, the model is trained using a dataset containing the metasurface structures and absorption spectra, and the trained model is capable of predicting the corresponding absorption spectra based on the given metasurface structure. Subsequently, the trained model is analyzed using the SHAP Deep Explainer to furnish both localized explanations for individual samples and comprehensive explanations across the entire dataset. With an intuitive visualization approach, we can better explain the following questions:

1. Which regions drive the model to make predictions?
2. How does each pixel contribute to the prediction?
3. For different metasurface structures, which channels contribute more to the prediction?

With the help of the SHAP Deep Explainer, we find the regions and channels that the model focuses on and calculate the SHAP value for each pixel point to determine their impact on the prediction. We find that besides the shape of the resonator, the model also focuses on parts other than the resonator, i.e., material properties. Moreover, the samples with hybrid dielectric structures pay more attention to the dielectric resonator’s thickness than the MIM structures. The detailed analysis is shown in the Supplementary Materials (Figures S11–S13).

This metasurface absorption spectra forward prediction framework works for any model and is not limited to our architecture. However, merely employing the SHAP Deep Explainer often results in speculative insights into the model’s predictions, lacking a clear and intuitive depiction of the specific features extracted from the SHAP value heatmap. The three problems explored here cannot provide an in-depth explanation of how neural networks work, rather they only investigate the model at the pixel level through the SHAP heatmap. Especially in the t channel, the model occasionally focuses on regions above the image without providing a logical explanation, potentially leading to confusion among designers. In addition, we found that the SHAP values in the neighboring pixels that the model focuses on are different in extent and positive and negative, a phenomenon that is difficult to explain. This is because each pixel is treated as an independent feature in this case, and the SHAP method is unable to take feature dependencies into account [38,47].

This pixel-level explanation is not in line with the human mindset, and it is impossible to ascertain whether the model understands the physical relationship between the metasurface structures and the absorption spectra. Moreover, this approach is unintuitive and requires careful observation to uncover the characteristics of the SHAP heatmap, which is not easy to use.

#### 3.3.2. Explanation at the Feature Level

To gain a deeper understanding of the working principle of the EP Network, we designed and trained the ER Network, which allows us to go beyond simply identifying the pixels the model focuses on. To further demystify the model’s operations, we used the EEPR framework to investigate the importance of each feature in the embedding vector rather than each pixel.

**Figure 5:**
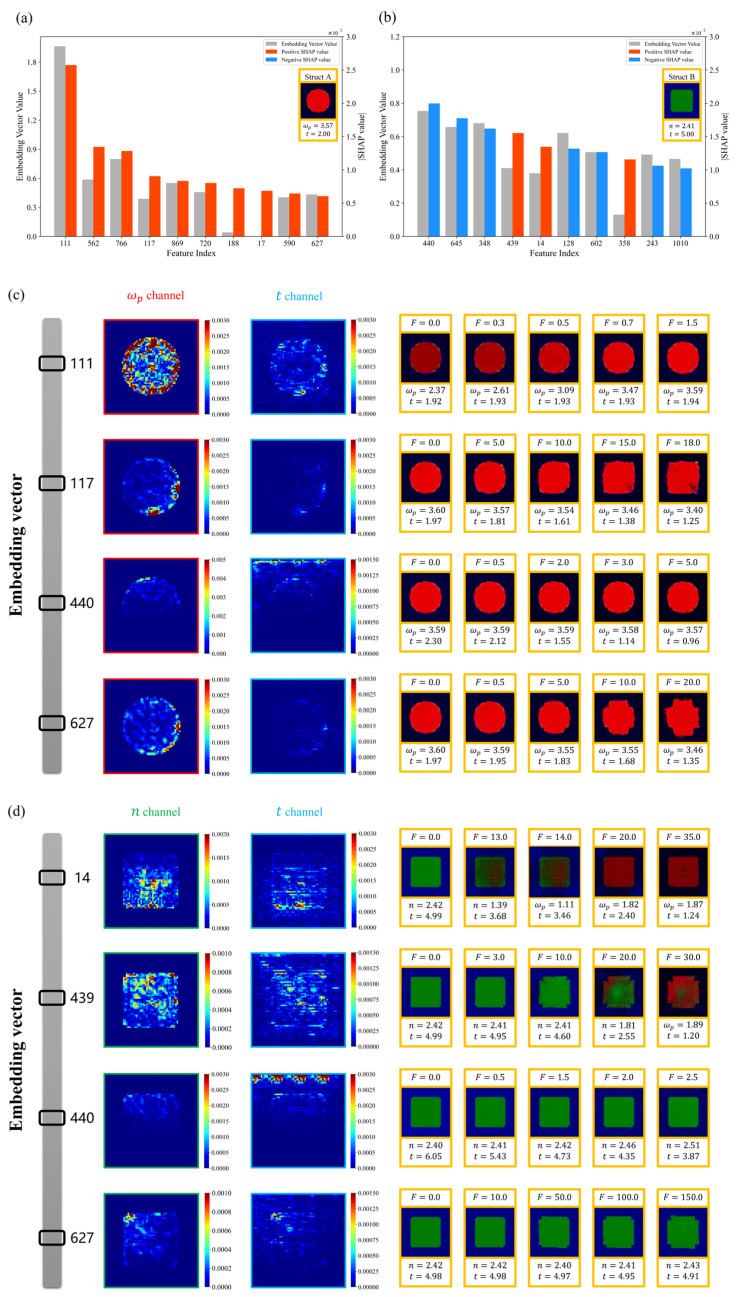
The meaning of the features extracted from the model and their effect on the structure: (**a**,**b**) show the ten dimensions with the largest absolute value of SHAP in the embedding vector of Struct A and Struct B, respectively; (**c**,**d**) show the absolute SHAP value heatmaps and the effect of modifying a dimension in the embedding vectors on Struct A and Struct B, respectively.

First, we used the SHAP deep explainer to obtain the SHAP values for each dimension (feature) in the embedding vectors. Figure 5a,b display the ten features with the largest absolute SHAP values for Struct A and Struct B, respectively. Given that SHAP values indicate feature importance, we generally focus on dimensions with larger absolute SHAP values for further exploration. Therefore, for Struct A, we selected Feature 111, Feature 117, and Feature 627. For Struct B, we selected Feature 14, Feature 439, and Feature 440. To explore the difference in the meaning of the features in the two types of structures, we also explored the effect of Feature 440 and Feature 627 in both Struct A and Struct B.

Subsequently, the E-part of the EP Network and the metasurface structures are input into the SHAP Deep Explainer to obtain the SHAP values for each pixel in the metasurface structures. By analyzing the heatmap of the absolute SHAP values and observing the effects of modifying the feature factors on the metasurface structure, we can identify the features extracted from a specific dimension of the embedding vector. Figure 5c,d present the heatmap of the absolute SHAP values and the effects on the structure caused by modifying the feature factor F for a specific dimension at the wave crest of Struct A and Struct B, respectively.

As shown in Figure 5b, Feature 111 is related to the plasma frequency of the metal resonator. As the plasma frequency increases, the value of Feature 111 also increases. The heatmap of the absolute SHAP values shows that the model primarily focuses on the inner region of the metal resonator, indicating that this feature predominantly captures the plasma frequency attribute. Feature 117 is associated with the edge of the metal resonator, and we find that it focuses on the degree of similarity between the resonator shape and the square by observing the structure change after adjusting the feature factor. Feature 440 focuses on the variation in the dielectric layer thickness, and smaller dielectric layer thickness results in a higher feature value, while it is insensitive to the variation in the shape of the resonator. An interesting phenomenon is that the model tends to extract the feature from the pixels in the upper region when obtaining the dielectric layer thickness, whereas we average all the pixels in the t channel to obtain this in the decoding process. This behavior may be attributed to the coding method: the resonator shapes of each sample in the dataset are varied, and the dielectric layer thickness information does not necessarily exist in the center region of the image, whereas the relevant information can always be extracted in the upper region. Additionally, the values of each valid pixel in the t channel are consistent in the dataset, leading the model to adopt a more straightforward way to extract the desired features. Feature 627 also focuses on the edge of the metal resonator, but unlike Feature 117, it focuses on the degree of similarity between the resonator shape and the cross. This indicates that the model not only extracts the edge features of the resonator but also makes predictions based on the degree of similarity between the resonator shapes of the current test sample and the samples in the training set.

As shown in Figure 5c, Feature 14 focuses on the material properties of the resonator and controls the type of the metasurface, which remains a hybrid dielectric structure when the feature factor is 13 and changes to a MIM structure when it is increased to 14. As the feature factor gradually increases, the metasurface gradually transforms from a hybrid dielectric structure to a MIM structure, while the shape of the resonator does not change significantly. Feature 439 focuses on multiple information. It can detect various changes in the type of the metasurface, material properties, thickness of the dielectric layer, and shape of the resonator at the same time. Similar to Struct A, Feature 440 in Struct B also focuses on the variation in the dielectric layer thickness and obtains dielectric layer thickness mainly from the top of the image. This indicates that Feature 440 can extract the thickness of the dielectric layer in both types of metasurface structures. In addition, Feature 627 can extract shape information in both the $\omega_{p}$ channel and the n channel regardless of the type of metasurface, and the same focus on the degree of similarity of the shape of the resonator to the cross shape is observed in the hybrid dielectric structure. Analyzing the interest region of this feature in the samples of both types of structures, we find that the model focuses on the lower right side of the resonator in Struct A and on the upper left corner of the resonator in Struct B, which further illustrates the model’s tendency to adopt a more straightforward way to extract the feature.

**Figure 6:**
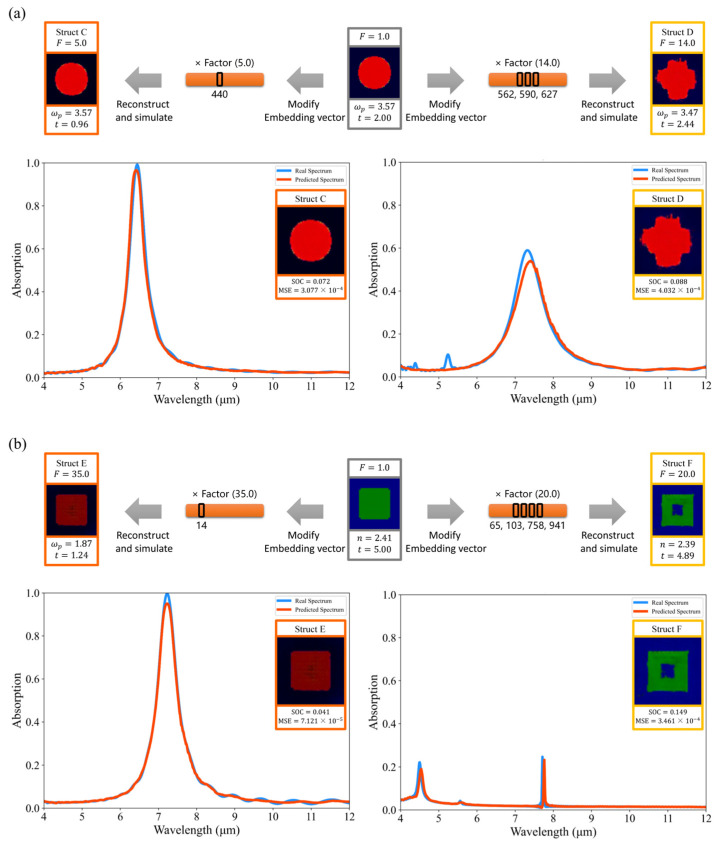
Absorption spectra of the metasurface structure obtained by modifying the embedding vector. (**a**) The embedding vectors of the MIM structure are modified to obtain Struct C and Struct D and simulations are performed to verify the predicted spectra. (**b**) The embedding vectors of the hybrid dielectric structure are modified to obtain Struct E and Struct F and simulations are performed to verify the predicted spectra.

In addition to adjusting the value of one of the dimensions in the embedding vector to explore the features extracted from that dimension, we can also adjust multiple dimensions simultaneously to explore the common effects of multiple features. For structure A, we selected three features that control the degree of similarity of the resonator shape to the cross: Feature 562, Feature 590, and Feature 627. In Figure 6a, adjusting the dimensions of the features (562, 590, and 627) that have an effect on the shape of the resonator at the same time can enhance the variation effect. For structure B, we randomly selected four features with small absolute SHAP values: Feature 65, Feature 103, Feature 758, and Feature 941. These features are not critical to the prediction of the current sample. However, this does not mean they do not affect the metasurface structure. In Figure 6b, adjusting the features in four dimensions (65, 103, 758, and 941) simultaneously can change the resonator’s shape from a solid square to a hollow square, which cannot be achieved by modifying any of the four feature dimensions alone. This indicates that the model is able to learn features that help predict the absorption spectra and that there may be some correlation between them, which are not necessarily all independent. The limitation of the SHAP method’s inability to take feature dependencies into account can be mitigated by observing the effects of multiple dimensions on the structure simultaneously.

**Figure 7:**
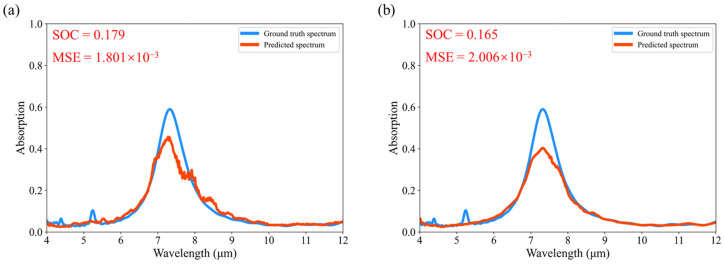
Predicted spectra and ground truth spectra of Struct D: (**a**,**b**) show the predicted spectra and ground truth spectra of UNet and ResNet, respectively.

In this way, we can gain a deeper understanding of what features are extracted by the model and on which features the model makes its predictions. Furthermore, our EP Network can accurately predict new metasurface structures derived from modifications to the embedding vectors, as illustrated in Figure 6. Even for structures significantly different from the training dataset, such as Struct D, our model maintains accurate predictions. In contrast, the predicted spectra from UNet and ResNet show less accuracy, as depicted in Figure 7. This indicates that our model can effectively extract the features of the structure and effectively set out to identify the effect of feature changes on the predicted spectra and learn the physical relationship between the metasurface structure and the absorption spectra to some extent.

Through feature-level explanations, we can not only analyze the meanings of the features and their effects on the metasurface structure but also draw the following conclusions:

1. The model tends to extract features in an easy and straightforward way.
2. The importance of features varies across different samples.
3. The model not only focuses on the edges of the resonator but also compares the similarity with the shape in the training data.
4. The features extracted by the model and the attributes in the dataset are not one-to-one correspondence, there may be some correlation between the features. The same feature may contain multiple attributes, and an attribute may also be controlled by multiple features.

**Figure 8:**
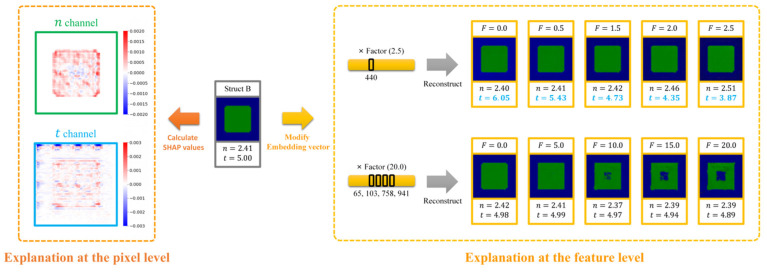
Comparison of feature-level explanation and pixel-level explanation.

Finally, to clearly illustrate the advantages of feature-level explanation over pixel-level explanation, we use Struct B as an example, as shown in Figure 8. Through pixel-level explanation, we obtain SHAP value images for the n channel and t channel, where each pixel represents a feature: red pixels indicate positive contributions, while blue pixels indicate negative contributions. Two issues arise with pixel-level explanation:

1. The model tends to focus excessively on the area above the *t* channel. Consequently, there is a possibility that the model has learned unreasonable rules, making it difficult for designers to trust the model.
2. In SHAP images, adjacent regions can have different pixel contributions, even though these pixel values are identical in the original image (even when they sometimes represent the same physical meaning). This is due to the limitations of SHAP in accounting for feature dependencies, resulting in phenomena that are difficult to understand.

In contrast, feature-level explanation provides a clearer understanding, as each dimension in the embedding vector represents a feature. Based on the previous analysis, we can see that Feature 440 focuses on the top edge region of the t channel to extract the dielectric thickness. The model does not consider the entire t channel because each pixel value in the t channel is identical in the dataset, and edge regions of the image are not occupied by the resonator in most cases. As a result, the model tends to extract features in a straightforward manner. Additionally, the EEPR framework can simultaneously analyze the impact of multiple features. As shown in Figure 8, increasing the values of Features 65, 103, 758, and 941 transitions the resonator from solid to hollow. This approach helps mitigate the limitations of SHAP’s inability to account for feature dependencies to some extent.

#### 3.3.3. Adjust Absorption at the Feature Level

**Figure 9:**
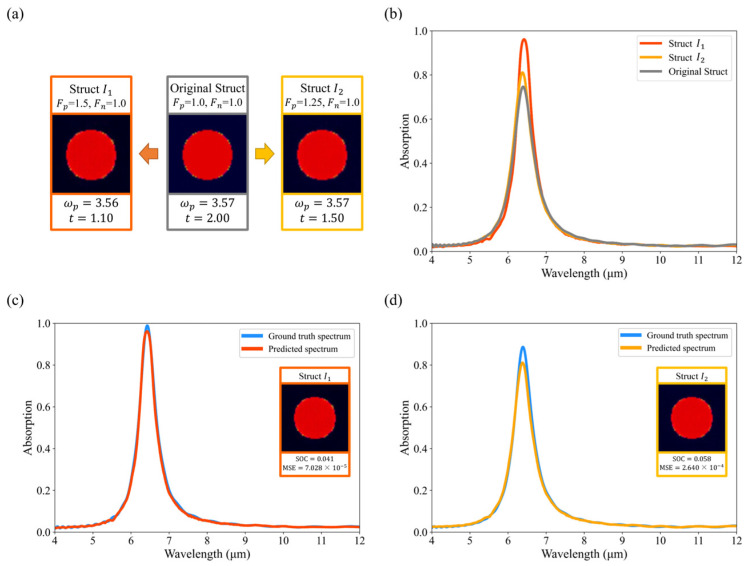
Adjust absorption by changing the original struct (Struct A) at the feature level. (**a**) Modify the embedding vectors of the original struct (Struct A) to obtain Struct $I_{1}$ and Struct $I_{2}$; (**b**) Absorption spectra of the original struct (Struct A), Struct $I_{1}$, and Struct $I_{2}$; (**c**,**d**) show the comparison of predicted spectra and ground truth spectra of modified structures.

**Figure 10:**
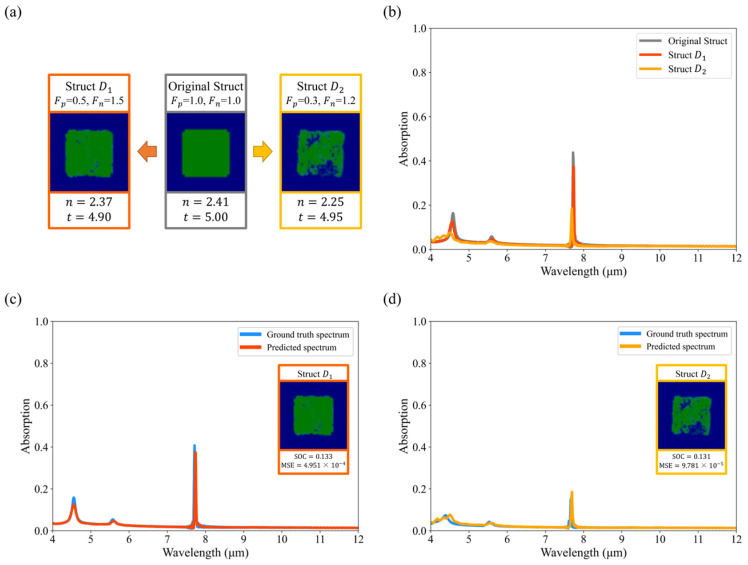
Adjust absorption by changing the original struct (Struct B) at the feature level. (**a**) Modify the embedding vectors of the original struct (Struct B) to obtain Struct $D_{1}$ and Struct $D_{2}$; (**b**) Absorption spectra of the original struct (Struct B), Struct $D_{1}$, and Struct $D_{2}$; (**c**,**d**) show the comparison of predicted spectra and ground truth spectra of modified structures.

With the EEPR framework, we can also modify the metasurface structure at the feature level to modulate the absorption values, as shown in Figure 9 and Figure 10. For both types of structures, the magnitude of the rise or fall of the absorption values can be controlled by adjusting the values of $F_{p}$ and $F_{n}$. To make the peak absorption of Struct A rise, the thickness of the dielectric layer needs to be reduced. As shown in Figure 9, the smaller the thickness, the higher the peak. Additionally, the number of pixels in the resonator pattern needs to be reduced, and the refractive index needs to be lowered to decrease the peak absorption of Struct B, as shown in Figure 10. Our method is able to visualize how the structure should be changed in order to make the absorption value at a particular wavelength go up or down, which can inspire designers to some extent, which is helpful when the metasurface is complex (e.g., when the resonator has a free-form shape instead of a regular shape).

The reconstructed structures were simulated using FDTD, and it was found that the EP Network’s prediction of them remained accurate. We found that slightly modifying multiple feature dimensions at the same time resulted in less drastic changes in the structure as compared to drastically modifying one feature dimension, allowing the modified structure to be as close as possible to the data distribution of the dataset and accurately predicting its absorption spectrum.

**Figure 11:**
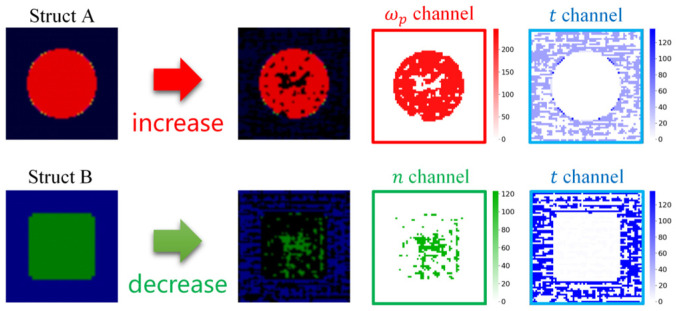
Pixel-level modifications on Struct A and Struct B.

Compared to the pixel-level explanation, we modify the embedding vectors instead of each pixel with the following advantages:

1. Our approach is independent of how the dataset is encoded. Under the conditions of image coding, there is an effective range of values for every pixel (e.g., 0~255), so we cannot adjust it freely. In addition, some pixel points have a value of 0 to represent the shape, which cannot be modified by multiplying a number. For example, since the hybrid dielectric structure has a value of 0 for all pixels in the *ω_p_* channel, it is impossible to change the structure type.
2. Our method is explainable, which can inspire designers to some extent, as shown in Figure 9 and Figure 10. To illustrate the advantages of modifying the metasurface at the feature level, we performed pixel-level modifications on the same metasurface, as shown in Figure 11. To modify the metasurface structure at the pixel level, removing all pixel points with positive SHAP values or all pixel points with negative SHAP values is common practice. The modified metasurface exhibits significant differences from the samples in the dataset, akin to randomly deleting parts of the pixels. This makes it challenging for designers to understand what physical properties should be altered to change the absorption values, and to discern actionable insights from the modified metasurface.

### 3.4. Ablation Study

The specific architecture that enhances the performance of the EP Network includes the following: (1) employing an Encoder–Decoder architecture to explicitly separate the prediction process into feature extraction and spectrum generation; (2) using pre-trained parameters from the ED Network; and (3) utilizing an adaptive pooling layer to compress the size of intermediate multi-channel feature maps to a 1 × 1 feature vector.

To evaluate the effectiveness of the Encoder–Decoder architecture in our task, we undertook a series of ablation studies. We removed the decoder part of the EP Network and directly output the predicted spectrum through a fully connected layer and a sigmoid activation function after the adaptive average pooling layer. For a fair comparison, we conducted the evaluation without using pre-trained parameters, as incorporating pre-training would further enhance prediction accuracy. After training for 500 epochs, the training loss was 0.031, and the average SOC and average MSE on the test set were 0.116 and $6.929\times 10^{-4}$, respectively. The comparison with the original model is shown in Table 2. Detailed information about the EP Network without the decoder part is provided in the Supplementary Materials (Figure S14).

The original model significantly outperforms the modified model in terms of both the SOC and MSE evaluation metrics on the test set. This demonstrates that using the Encoder–Decoder architecture can effectively improve prediction accuracy. Additionally, although the modified model has fewer parameters, it exhibits more severe overfitting. This indicates that the modified model has not adequately learned the physical relationship between the metasurface and the absorption spectra. The Encoder–Decoder architecture separates the prediction process into feature extraction and spectra generation, compelling the network to extract features within the metasurface that are useful for spectral prediction. This approach better captures the physical relationship between metasurface structures and absorption spectra, thereby enhancing prediction accuracy and reducing overfitting.

To ensure that the model extracts features before prediction, we first train the ED Network so that the E-part effectively extracts features, encoding the high-dimensional metasurface structure into a low-dimensional embedding vector. Subsequently, we transfer the parameters of the E-part from the ED Network to the EP Network. We set the learning rate to 0.0006 for the E-part and 0.001 for the P-part during the training of the EP Network. In this case, the average SOC of the EP Network on the test set is 0.079, and the average MSE is 2.843$\times 10^{-4}$, as shown in Table 3. Based on the results in the table, we can conclude the following:

- When the EP Network inherits the parameters from the E-part and continues training with appropriate learning rates for both the E-part (0.0006) and P-part (0.001), the SOC decreases by 5.95% and the MSE decreases by 5.51%. This suggests that enforcing feature extraction before prediction in this manner is effective. It not only facilitates network explainability after training but also enhances network performance.
- Using the E-part of the ED Network as the initial parameters alone does not lead to significant improvements and may even result in an increase in the MSE. This indicates that pre-training enhances accuracy by clearly delineating the distinct roles of feature extraction and spectra generation between the E-part and P-part. Consequently, a smaller learning rate should be applied to the E-part to preserve its function.
- Freezing the E-part parameters after inheriting them from the ED Network can lead to a substantial decline in performance. This implies that the encoding approach of the ED Network is a lossy compression not tailored for absorption spectra prediction tasks, resulting in the loss of crucial information. Consequently, fine-tuning of the E-part is required to retain its effectiveness.

**Table 3:** Performance comparison of different pre-trained methods.

Pre-Train	Learning Rate of E-Part	Learning Rate of P-Part	Test Loss (SOC)	Test Loss (MSE)
N	0.001	0.001	0.084	$3.009\times 10^{-4}$
Y	0.001	0.001	0.082	$3.053\times 10^{-4}$
Y	0	0.001	0.091	$3.896\times 10^{-4}$
Y	0.0006	0.001	0.079	$2.843\times 10^{-4}$

Note: Details of the learning rate adjustments are provided in Table S6.

To enhance explainability and training efficiency, we incorporated an adaptive average pooling layer in the middle of the network to compress multi-channel feature maps into embedding vectors. To evaluate the effectiveness of this design, we removed the adaptive average pooling layer. Additionally, we investigated the impact of using multi-channel feature maps (1024 × 2 × 2) instead of embedding vectors. Detailed information about the EP Network without an adaptive average pool and the EP Network with a multi-channel feature map is provided in the Supplementary Materials (Figures S15 and S16). The results are summarized in Table 4, showing the following findings:

- Comparison with the EP Network without the adaptive average pool: Using embedding vectors reduces the SOC by 4.81%, the MSE by 12.14%, and the training time by 24.59% compared to not using embedding vectors. This indicates that embedding vectors force the network to extract more useful information, suggesting that using embedding vectors as a bridge between the E-part and P-part helps the E-part to better extract features and store them in a lower-dimensional vector, which is then utilized by the P-part. Additionally, this approach reduces the data volume handled by the first transposed convolutional layer of the P-part, leading to shorter training times.
- Comparison with the EP Network with the multi-channel feature map: Using embedding vectors instead of multi-channel feature maps reduces the SOC by 3.65%, the MSE by 12.06%, and the training time by 37.18%. This demonstrates that embedding vectors, as opposed to multi-channel feature maps, decrease the number of network parameters, thereby improving training efficiency and prediction accuracy.

**Table 4:** Performance comparison with or without the embedding vectors.

Model	Params Size (MB)	Training Time (Seconds)	Test Loss (SOC)	Test Loss (MSE)
EP Network without an adaptive average pool	115.710	$1.436\times 10^{4}$	0.083	$3.236\times 10^{-4}$
EP Network with multi-channel feature map	203.740	$1.724\times 10^{4}$	0.082	$3.233\times 10^{-4}$
EP Network	175.710	$1.083\times 10^{4}$	0.079	$2.843\times 10^{-4}$

Note: All listed models can utilize the pre-trained parameters of the ED Network. Since the ED Network only needs to be trained once, the training time for the ED Network is not considered here.

In summary, the Encoder–Decoder architecture is the foundation for using pre-trained parameters from the ED Network. At the same time, using the Encoder–Decoder architecture increases the model’s parameter count, which may lead to increased training time. As compensation, the use of embedding vectors enhances training efficiency, offsetting the potential increase in training time due to the increased model complexity to some extent. To explore the limitations of the model, we conducted a further investigation into the loss function (Figure S17) and samples in the datasets (Figure S18).

## 4. Conclusions

In this study, we introduced the EEPR framework, the first user-friendly, explainable framework specifically designed for predicting metasurface absorption spectra. This novel approach employs an Encoder–Decoder architecture within the EP Network, effectively segregating the prediction process into distinct phases of feature extraction and spectral generation. By leveraging the unique architecture of the EP Network and pre-trained parameters from the ED Network, our framework not only achieves high performance with an average SOC of 0.079 and an MSE of 2.843 $\times \;10^{-4}$ on the test set, but also significantly reduces prediction times to just $3\times 10^{-3}$ seconds, which is approximately 500,000 times faster than the FDTD simulation. Furthermore, the integration of the ER Network and SHAP heatmaps allows for a deeper exploration of the black box of AI models at a feature level, enhancing explainability and trust among designers. Our approach facilitates a more intuitive understanding of AI predictions by enabling the visualization of feature influences on metasurface structures and offers the flexibility to analyze the effects of multiple features simultaneously. This capability addresses the limitations of existing SHAP applications, which do not account for feature dependencies, and presents a scalable solution across different models and electromagnetic responses.

However, due to the difficulty of data collection for metasurfaces, we tested it on an open-source dataset containing only two metasurface types. In this dataset, only plasma frequency, refractive index, and dielectric layer thickness are considered in addition to the shape of the resonator. There are only three possible values for both the plasma frequency and the dielectric layer thickness in MIM structures, and only three are for refractive index and dielectric layer thickness in hybrid dielectric structures. Therefore, the number of features we can analyze is limited. Although we can adjust the absorption by modifying structures at the feature level as needed, the model may not accurately predict the spectra when the structures change significantly. This may be due to insufficient diversity of training samples.

In the future, we plan to collect a dataset containing more attributes. Due to the challenges in data collection, we plan to explore the application of transfer learning for predicting electromagnetic responses of nanophotonic devices, aiming to reduce the reliance on large datasets. Based on this, we will further improve the model’s performance and explore the meaning of the extracted features.

## Figures and Tables

**Table 1:** Model performance comparison.

Model	Training Loss (SOC)	Test Loss (SOC)	Test Loss (MSE)
FullyConnectedNet [18]	0.125	0.155	$1.177\times 10^{-3}$
CNN [13]	0.152	0.154	$1.064\times 10^{-3}$
CapsNet [44]	0.093	0.141	$1.123\times 10^{-3}$
VIT [45]	0.064	0.110	$5.540\times 10^{-4}$
UNet [46]	0.049	0.117	$5.190\times 10^{-4}$
ResNet [16]	0.031	0.108	$6.158\times 10^{-4}$
EP Network	0.054	0.084	$3.009\times 10^{-4}$
EEPR framework	0.047	0.079	$2.843\times 10^{-4}$

Note: Without considering the EEPR framework, the EP Network alone achieves good accuracy, with a test set SOC of 0.084 and an MSE of 3.009 $\times 10^{-4}$. Incorporating the EP Network into the EEPR framework, which utilizes pre-trained parameters from the ED Network, further enhances the performance of the EP Network, achieving a test set SOC of 0.079 and an MSE of 2.843$\times 10^{-4}$. At this point, the accuracy of the EP Network can be considered representative of the accuracy of the entire framework.

**Table 2:** Performance comparison before and after removing the decoder part of the EP Network.

Model	Params Size (MB)	Training Loss (SOC)	Test Loss (SOC)	Test Loss (MSE)
EP Network without decoder part	74.580	0.031	0.116	$6.929\times 10^{-4}$
EP Network	175.710	0.054	0.084	$3.009\times 10^{-4}$
EEPR framework	175.710	0.048	0.079	$2.843\times 10^{-4}$

Note: Here, the EEPR framework refers to the EP Network utilizing the pre-trained parameters from the ED Network.

## Data Availability

The original contributions presented in this study are included in the article/Supplementary Materials. Further inquiries can be directed to the corresponding author.

## Supplementary Materials

The following supporting information can be downloaded at: https://www.mdpi.com/article/10.3390/nano14181497/s1, Figure S1: Architecture of the ER network; Figure S2: Details of the model architecture; Figure S3: Illustration of SHAP value contributions; Figure S4: Diagram of feature-level explanation; Figure S5: Diagram of Modifying the Metasurface at the Feature Level; Figure S6: Learning curve of EP Network; Figure S7: Learning curve of ED Network; Figure S8: Learning curve of ER Network; Figure S9: Schematic diagram of metasurfaces structures and encoding; Figure S10: Case study of the model’s generalization ability; Figure S11: Areas of interest for model; Figure S12: Analysis of the reasons why the model makes predictions; Figure S13: SHAP Global explanations on image channels; Figure S14: Architecture of the EP Network without Decoder part in Section 3.4; Figure S15: Architecture of the EP Network without adaptive average pool in Section 3.4; Figure S16: Architecture of the EP Network with multi-channel feature map in Section 3.4; Figure S17: Histogram of loss distribution; Figure S18: Limitations of model prediction for absorption spectra;Table S1: Comparison of using SOC and MSE as the loss function; Table S2: Parameter sizes and training times of the models listed in Table 2; Table S3: List of hyperparameter and their values; Table S4: Approximate memory requirements of the MIM structures; Table S5: Approximate memory requirements of the hybrid dielectric structures; Table S6: Test Losses with Varying Learning Rates for E-part.
