# Supplementary material for: Explainable Encoder–Prediction–Reconstruction Framework for the Prediction of Metasurface Absorption Spectra
Source: Nanomaterials (Basel). 2024 Sep 14;14(18):1497. doi: 10.3390/nano14181497 (PMC11434424; doi:10.3390/nano14181497)
Supplement: Supplementary file 1 [file nanomaterials-14-01497-s001.zip › nanomaterials-3158895-supplementary.pdf]

Supplementary Material of

Explainable Encoder–Prediction–Reconstruction Framework for the prediction of Metasurface Absorption Spectra

Yajie Ouyang [1], Yunhui Zeng [2,*] and Xiaoxiang Liu [1]

[1] School of Intelligent Systems Science and Engineering, Jinan University, Zhuhai 519070, China; oyyj2003@outlook.com (Y.O.); tlxx@jnu.edu.cn (X.L.)
[2] Shenzhen International Graduate School, Tsinghua University, Shenzhen 518055, China;
* Correspondence: zengyh22@mails.tsinghua.edu.cn (Y.Z.)

1. Model architecture

The E-part of the ER Network is identical to the EP network, with the input and output as metasurface structures (3×64×64 RGB images), and MSE is used as the loss function to reconstruct the input metasurface structures. The architecture is shown in Figure S1. The ED Network aims to obtain a low-dimensional vector representation of the metasurface structure, providing better initialization parameters for the EP network. Therefore, the Encoder part needs to be consistent with the E-part of the EP Network. The ER Network is divided into the E-part and the R-part. Its purpose is to reconstruct the embedded vector into a metasurface structure. Therefore, its E-part also needs to maintain the same network architecture as the E-part of the EP Network and freeze the parameters so that the R-part can be used to reconstruct the metasurface structure encoded by the embedding vector in the EP Network. The ED Network and ER Network share similar architectures, the only difference being the presence or absence of the Tanh activation function.

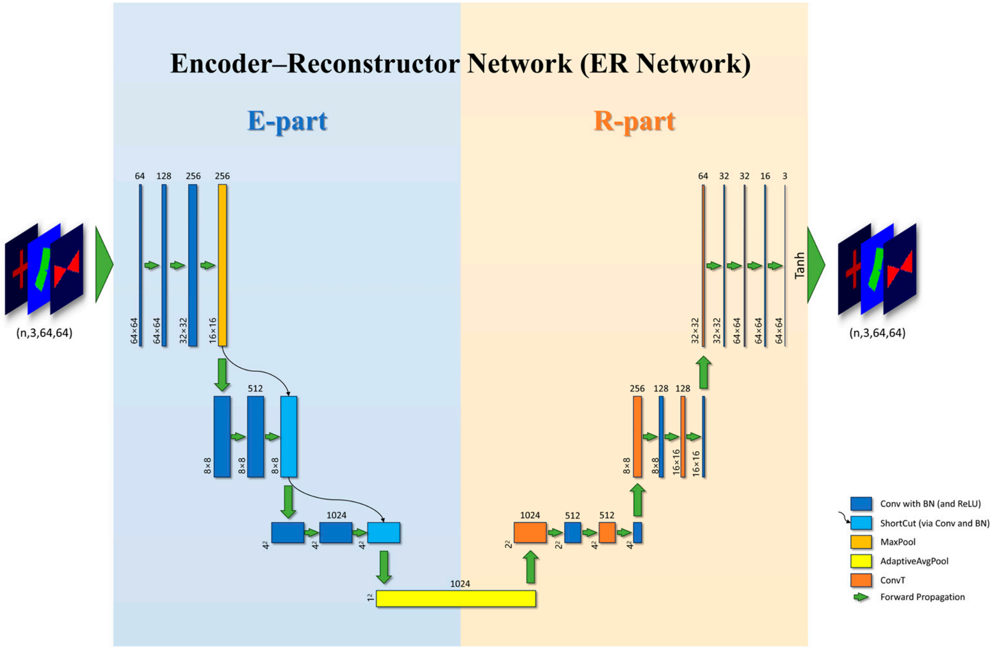

Figure S1. Architecture of the ER network.

Figure S2 illustrates the detailed architecture of the model. Notably, the E-part architecture is consistent across all three networks, and the architectures of the E-part, P-part, and R-part are circled using green, yellow, and orange dashed lines, respectively.

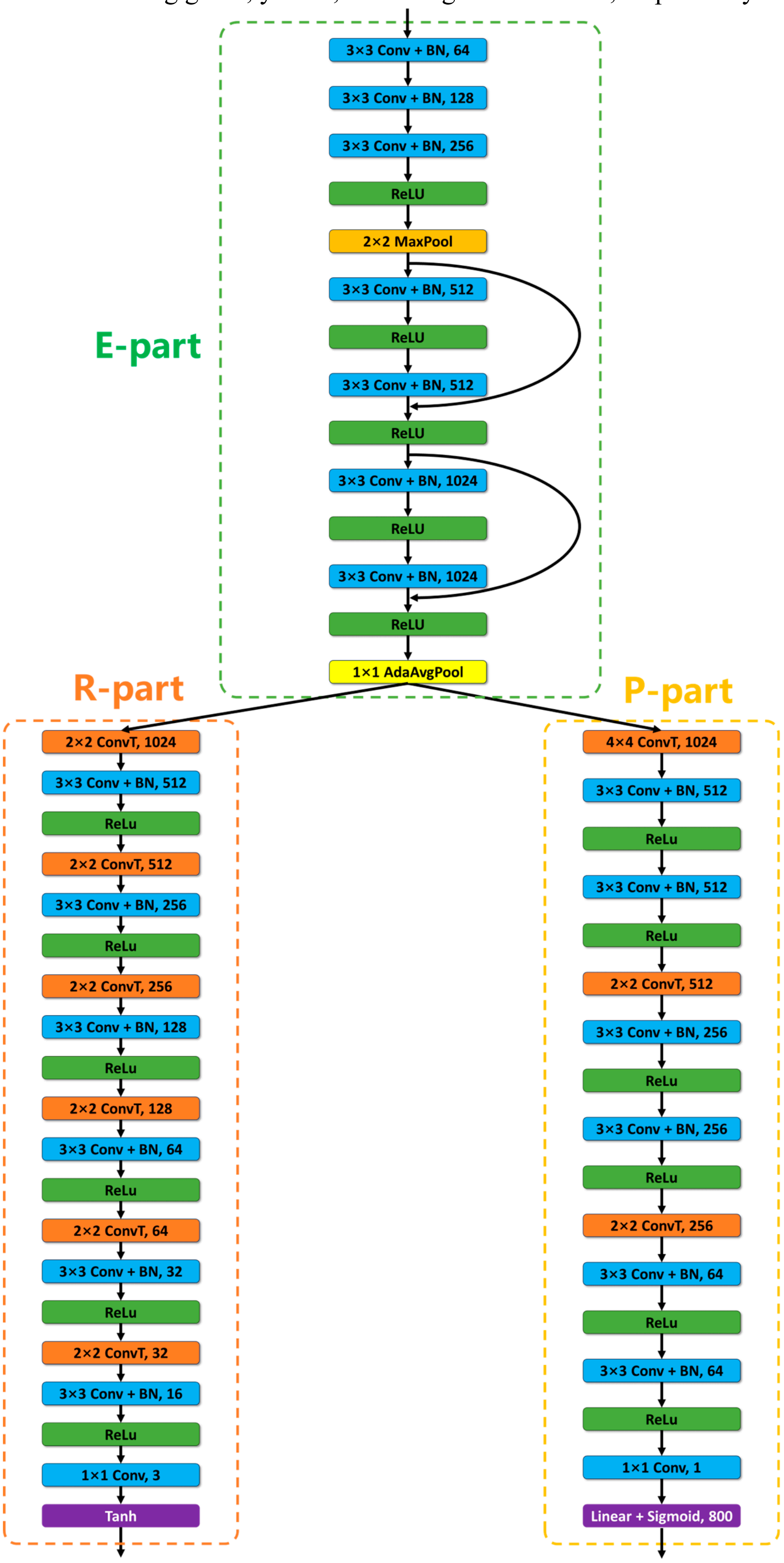

Figure S2. Details of the model architecture.

2. Spectral overlap coefficient as the loss function

To demonstrate the advantages of SOC compared to MSE, we conducted experiments on the models listed in Table 1, with results presented in Table S1. For each model, only the loss function was different, while other conditions, such as hyperparameters and training epochs, were kept constant.

Table S1. Comparison of using SOC and MSE as the loss function.

Model	Loss function	Test loss (SOC)	Test loss (MSE)
FullyConnectedNet	SOC	0.155	1.177×10^{-3}
FullyConnectedNet	MSE	1.000	2.250×10^{-2}
CNN	SOC	0.154	1.064×10^{-3}
CNN	MSE	1.000	2.250×10^{-2}
CapsNet	SOC	0.141	1.123×10^{-3}
CapsNet	MSE	0.167	9.882×10^{-4}
VIT	SOC	0.110	5.540×10^{-4}
VIT	MSE	1.000	2.250×10^{-2}
UNet	SOC	0.117	5.190×10^{-4}
UNet	MSE	1.000	2.250×10^{-2}
ResNet	SOC	0.108	6.158×10^{-4}
ResNet	MSE	0.127	6.174×10^{-4}
EP Network	SOC	0.084	3.009×10^{-4}
EP Network	MSE	0.108	3.889×10^{-4}

The results in Table S1 highlight the following benefits of SOC:

Effective Optimization: The SOC loss function demonstrates superior performance in optimizing the target, effectively minimizing both SOC and MSE. Across all models, the test loss (SOC) was consistently lower when SOC was employed as the loss function. Notably, in most cases, using SOC instead of MSE resulted in a lower MSE on the test set. For example, in our model, replacing MSE with SOC as the loss function led to a 22.62 % reduction in MSE on the test set.

Adaptability and Stability: The SOC loss function shows greater adaptability and stability across various model architectures, whereas MSE may result in model collapse. Models like FullyConnectedNet, CNN, VIT, and UNet experienced collapses when MSE was used as the loss function. In contrast, under the same hyperparameter settings, all models performed better when SOC was used as the loss function. This suggests that MSE is more sensitive to hyperparameters, requiring different hyperparameter settings for optimal performance across different network architectures. Adjusting hyperparameters during neural network training requires expertise and can be time-consuming, making MSE less favorable due to its complexity in tuning. Conversely, SOC is easier to configure and more user-friendly, especially for practitioners outside the AI field.

However, it is important to note that MSE remains the most widely used loss function. In existing work on absorption spectra prediction, MSE is predominantly used as the evaluation metric. Therefore, while SOC is superior during training, MSE remains the better choice for performance comparison.

For further insights into the advantages of using SOC as a loss function for spectral prediction tasks, please refer to our previous work[1].

3. Supplementary explanation of the EEPR framework

For a machine learning model, the sum of all Shapley values[2–4] and the average predicted value is equal to the prediction, as explained in more detail in Figure S3. The figure demonstrates the additive nature of SHAP values, which is one of the fundamental properties of SHAP. For example, if the model extracts seven features, each contributing as shown in the figure—where red represents positive contributions and blue represents negative contributions—the expected output from the background dataset is 0.59. By adding the contribution of each feature, the model produces an output of 0.87 for this test sample. Due to the high computational cost of SHAP values, we typically use Python packages[3] to approximate SHAP values in engineering practice.

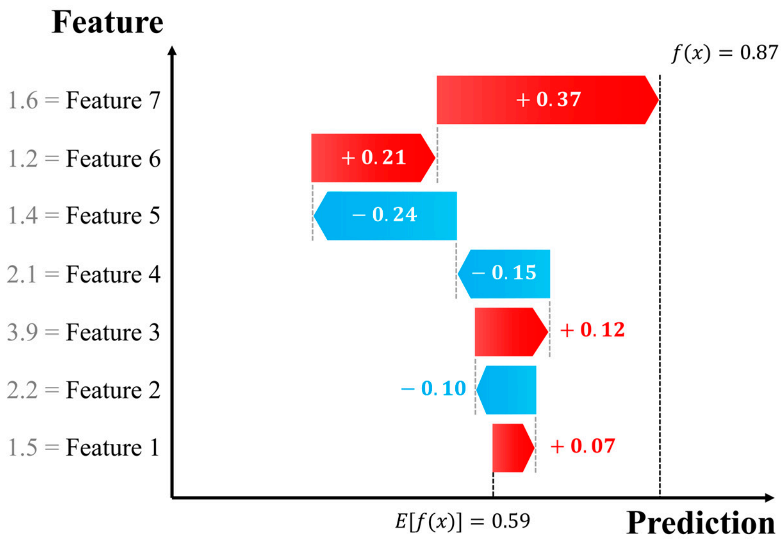

Figure S3. Illustration of SHAP value contributions.

Figure S4 illustrates the process of feature-level explainability. First, the metasurface is input into the E-part, generating an embedding vector. The desired features are then adjusted and input into the R-part, allowing the exploration of how these features impact the metasurface. The formulas shown in the figure have been explained in the main text.

Figure S5 illustrates the process of modifying metasurface at the feature level to adjust absorption values. After obtaining the embedding vector, the SHAP Deep Explainer is used to calculate the SHAP values for each feature. Based on the sign of the SHAP values, the features are divided into two groups, which are then multiplied by positive and negative feature factors, respectively. The modified embedding vector is subsequently input into the R-part, which generates the corresponding metasurface. By adjusting the value of the positive and negative feature factors, the extent of the absorption value change can be controlled. The formulas shown in the figure have been explained in the main text.

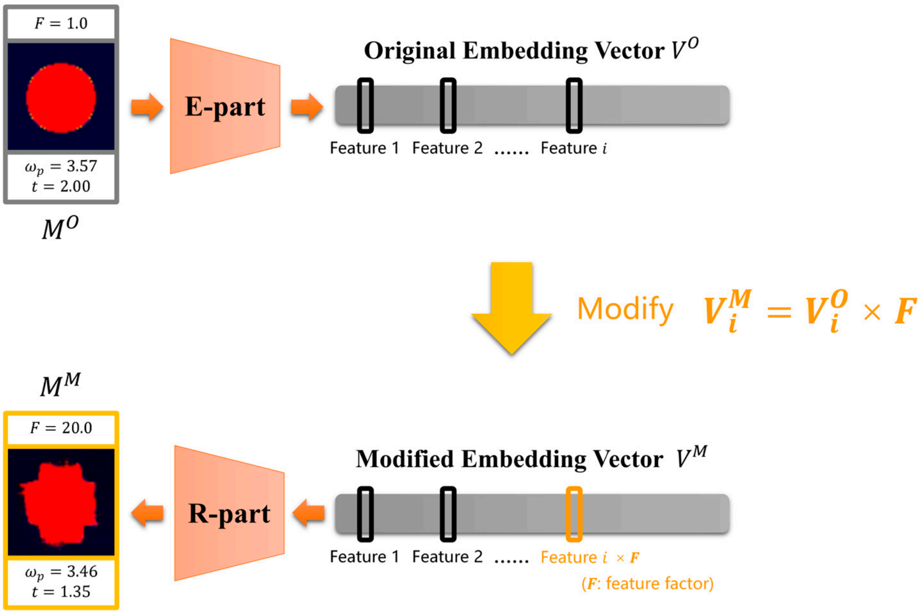

Figure S4. Diagram of feature-level explanation.

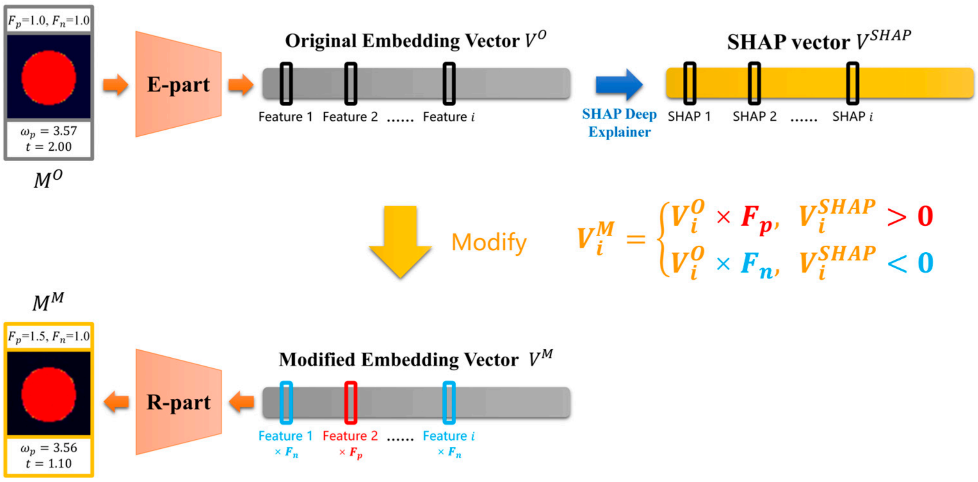

Figure S5. Diagram of Modifying the Metasurface at the Feature Level.

4. Model training

Figure S6 presents the learning curve of the EP Network. Figure S7 presents the learning curve of the ED Network. After 500 epochs of training, the loss on the training set is 4.282×10^{-4}, and the loss on the test set is 5.044×10^{-4}. Figure S8 presents the learning curve of the ER Network. After training for 500 epochs, the loss on the training set is 1.710×10^{-4}, and the loss on the test set is 2.743×10^{-4}.

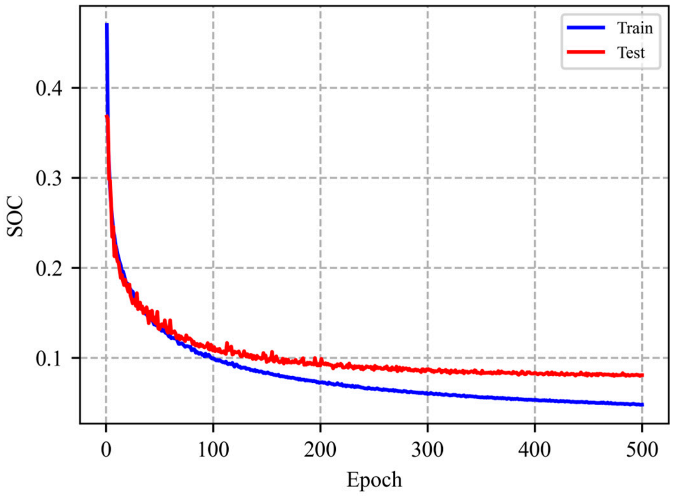

Figure S6. Learning curve of EP Network.

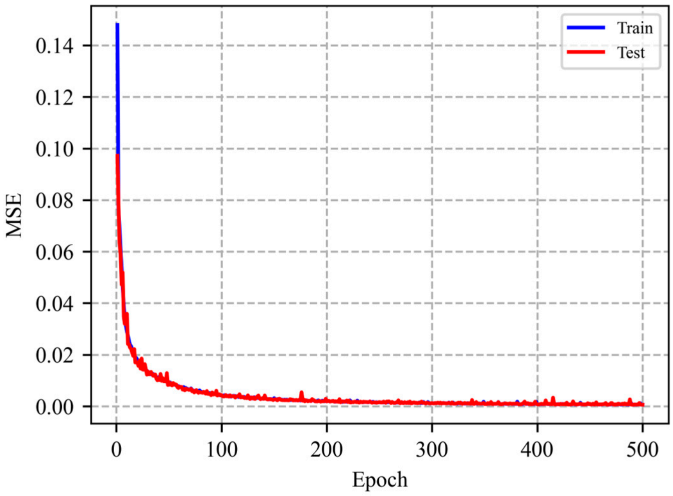

Figure S7. Learning curve of ED Network.

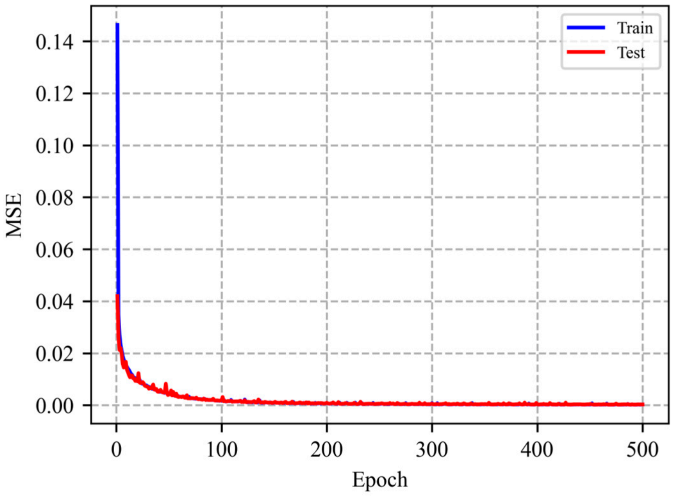

Figure S8. Learning curve of ER Network.

5. Dataset

In this dataset[5], the structures of the metasurfaces are represented by three-channel RGB images with two classes of absorbing metasurfaces: metal-insulator-metal (MIM) structures and hybrid dielectric structures. These two types of metasurfaces were chosen for realistic applications: the MIM structures have a relatively broad Lorentzian shaped absorption response, which is suitable for thermal emission and energy harvesting applications, as shown in Figure S9 (e). The hybrid dielectric structures utilize the cavity effect to produce asymmetric narrow-band Fano resonance, which is suitable for optical sensing and detection applications, as shown in Figure 9 (f). The MIM-structured type of the metasurface is shown in Figure S9 (a), with a thin dielectric layer on the bottom metal layer, and on top of the dielectric layer is a metal freeform resonator (lithographically patterned), encoded as shown in Figure S9 (c), using the R channel for the freeform resonators shape and material properties and the B channel for the dielectric thickness. The hybrid dielectric structure metasurface is shown in Figure S9 (b), with a metal film substrate on the bottom and a dielectric freeform resonators on top, encoded as shown in Figure S9 (d), using the G channel for the shape and material properties of the dielectric resonators and the B channel for the thickness of the dielectric resonators. For convenience, we denote the R channel, G channel, and B-channel as the plasma frequencies channel (ω_p channel), the refractive indices channel (n channel), and the dielectric thicknesses channel (t channel), respectively. The shape pattern is generated by seven shape templates, including cross, square, ellipse, bow-tie, H, V, and tripole-shaped, with the size of 64 × 64 pixels, which can be freely rotated and stretched.

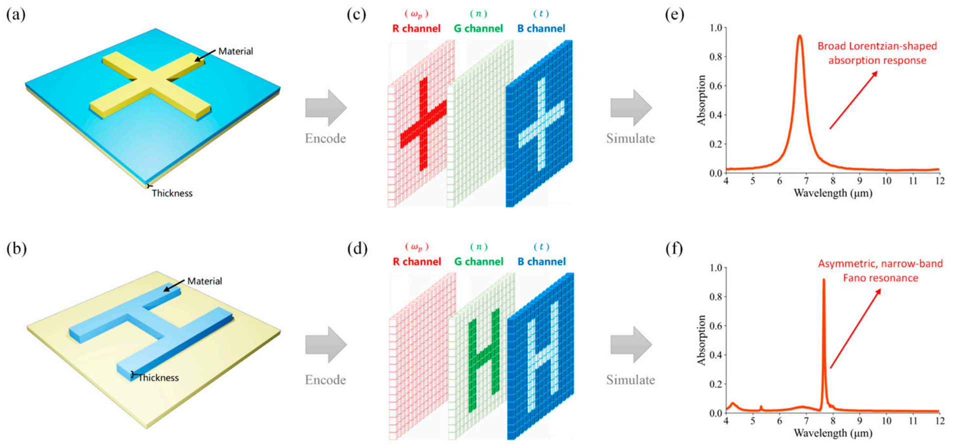

Figure S9. Schematic diagram of metasurfaces structures and encoding. (**a**) and (**b**) show the structural diagrams of the MIM structure and the hybrid dielectric structure, respectively. (**c**) and (**d**) show the encoding diagrams of the MIM structure and the hybrid dielectric structure, respectively. (**e**) and (**f**) show the absorption spectra of the MIM structure and the hybrid dielectric structure, respectively.

6. Detail of Experiment

The parameter sizes and training times for the models listed in Table 1 are shown in Table S. In the EEPR framework, the EP network utilizes pre-trained parameters from the ED network, which contributes to the overall longer training time. However, it is important to note that the ED network only needs to be trained once. Once trained, these parameters can be used across various hyperparameter settings for the ED network and even for networks with architectures similar to the EP network. Additionally, the training time for VIT is significantly longer, approximately 2.2 times that of our model (including pre-training time). This extended training duration may be attributed to the high computational complexity associated with the self-attention mechanism used in VIT.

Table S2. Parameter sizes and training times of the models listed in Table 2.

Model	Params size (MB)	Training time (seconds)
FullyConnectedNet	556.880	6.464×10^3
CNN	2.680	6.414×10^3
CapsNet	19.150	2.185×10^4
VIT	6.440	4.895×10^4
UNet	81.420	1.154×10^4
ResNet	73.310	8.768×10^3
EP Network	175.710	1.083×10^4
EEPR framework	175.710	2.266×10^4

The results presented in Table 1 were obtained under the same hyperparameter conditions detailed in Table S3. Unless otherwise specified, all experimental results reported in this paper were obtained under these hyperparameter conditions. In deep learning, setting the learning rate to 0.001 and using the Adam optimizer are common practices that work well for most tasks. The batch size is typically chosen from values such as 2, 4, 8, 16, 32, 64, 128, 256, etc., and should be set according to hardware capabilities, generally being the largest size that can be supported by the hardware. The number of training epochs should be set according to the specific task to ensure that the model converges effectively.

Table S3. List of hyperparameter and their values.

Hyperparameter	Value
Learning rate	0.001
optimizer	Adam
Mini-batch size	64
Number of training epoch	500

In our experiments, we used Python version 3.11, PyTorch version 2.2.0+cu121, Lumerical FDTD version 2020 R2, and SHAP version 0.42.

7. Detail of FDTD simulation

We used Lumerical FDTD (a commercial package) for simulation, version 2020R2. Before running the simulations, it is essential to check the approximate total memory

requirements to avoid swapping during the simulation process. The approximate memory required for the MIM structures and the hybrid dielectric structures is provided in Tables S4 and S5, respectively.

Table S4. Approximate memory requirements of the MIM structures.

**Stage**	**Memory requirement**
Initialization and mesh	82.000 MB
Running simulation	1.623 GB
Data collection	2.599 GB
Monitor data saved to fsp file	2.000 MB
Total	4.306 GB

Table S5. Approximate memory requirements of the hybrid dielectric structures.

**Stage**	**Memory requirement**
Initialization and mesh	82.000 MB
Running simulation	2.562 GB
Data collection	3.575 GB
Monitor data saved to fsp file	2.000 MB
Total	6.221 GB

The experiments were conducted on a machine equipped with an Intel(R) Core(TM) i7-14700KF CPU and a single NVIDIA GeForce RTX 4090D GPU. On this setup, it took an average of 5 minutes per sample for the MIM structures and 45 minutes per sample for the hybrid dielectric structures at peak performance. In contrast, using our framework, the absorption spectra of metasurfaces can be predicted in batches. With a batch size of 64, it only takes an average of 3×10^{-3} seconds per sample.

8. Case Study

In practical applications, the model's generalization ability is crucial. To illustrate the generalizability of our model, we conducted a case study by designing three resonator shapes—ring, hexagon, and pentagon—which were not included in the dataset, as shown in Figure S10 (a). The ring-shaped resonator is made of aluminum, and the hexagon-shaped resonator is made of silver; both are MIM structures with a dielectric layer thickness of 0.1 μm. The pentagon-shaped resonator is made of silicon with a thickness of 0.5 μm, and it belongs to the hybrid dielectric structures. After encoding these three metasurfaces, we used the four models with the lowest loss on the test set to predict the absorption spectra of them. Figure S10 (b), (c), (d), and (e) show the prediction results from our model, VIT, ResNet, and UNet, respectively.

As observed, our model can accurately predict the spectra of metasurfaces that differ significantly from the training data. In contrast, the prediction accuracy of VIT noticeably declines, and ResNet and UNet even produce artifacts in their predictions, clearly struggling to handle samples that diverge substantially from the training set. In this context, "artifacts" refers to unintended or irregular features in the predicted absorption spectra, such as jagged or spiky patterns, that do not correspond to the expected smooth spectrum. These artifacts indicate that the model is having difficulty making accurate predictions for these particular samples. Overall, our

model demonstrates superior generalization and practicality compared to other mainstream networks.

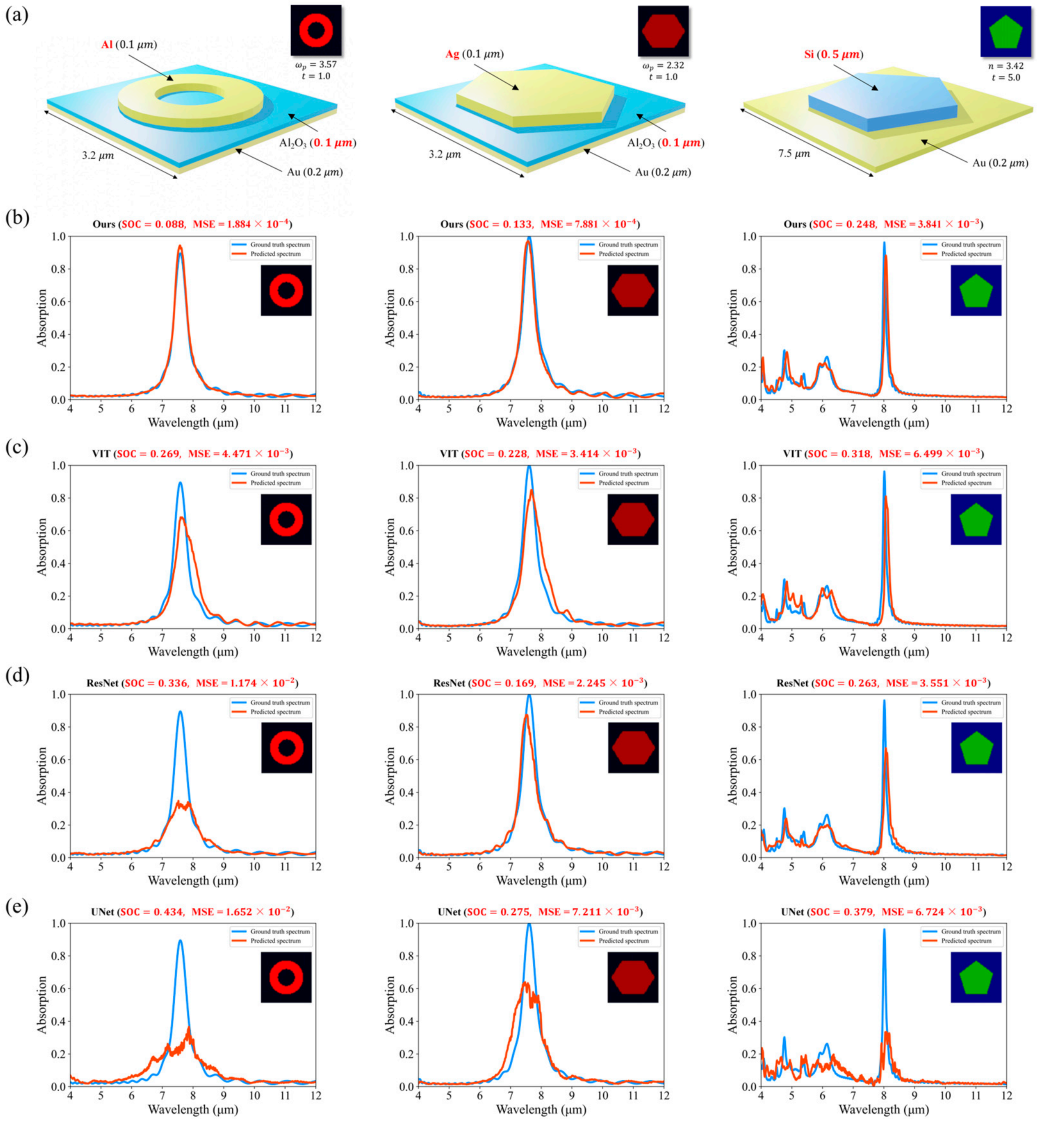

Figure S10. Case study of the model's generalization ability. (a) Metasurfaces that differ significantly from the samples in the dataset. (b) Prediction results from our model. (c) Prediction results from VIT. (d) Prediction results from ResNet. (e) Prediction results from UNet.

9. Detail of Explanation at the pixel level

We input the trained EP Network and the RBG images representing the metasurfaces structures into the SHAP Deep Explainer to compute the SHAP values. To identify which regions influenced the model to make predictions for a particular sample, we considered all wavelengths and all channels and presented the mean absolute value of SHAP as a visualization, as shown in Figure S11. The figure highlights the regions of focus for the model across four samples. The model not only focuses on the edges of the resonator but also the interior has a higher SHAP value because the interior encodes the material properties of the

resonator, which suggests that the model not only focuses on the shape of the free resonator but also focuses on the material property information. In the third and fourth samples, the model also focuses on regions beyond the resonator, indicating that it is extracting the thickness of the dielectric resonator. In addition, the model also focuses on the hollow part in the middle of the square in the second sample, which indicates that the solid and hollow centers will influence the model prediction. The model can focus on the critical regions for different types of metasurfaces and resonator shapes and thus give accurate predictions.

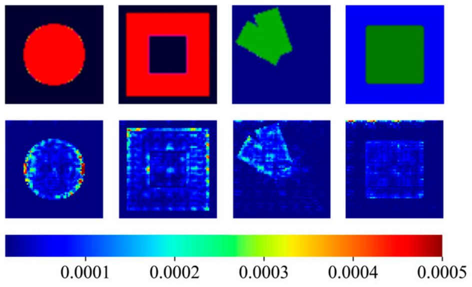

Figure S11. Areas of interest for model.

To further analyze why the model makes predictions and to explore how each pixel point contributes to the prediction, we present the SHAP values of the three channels at the wavelength where the peak is located in the form of images, as shown in Figure S12.

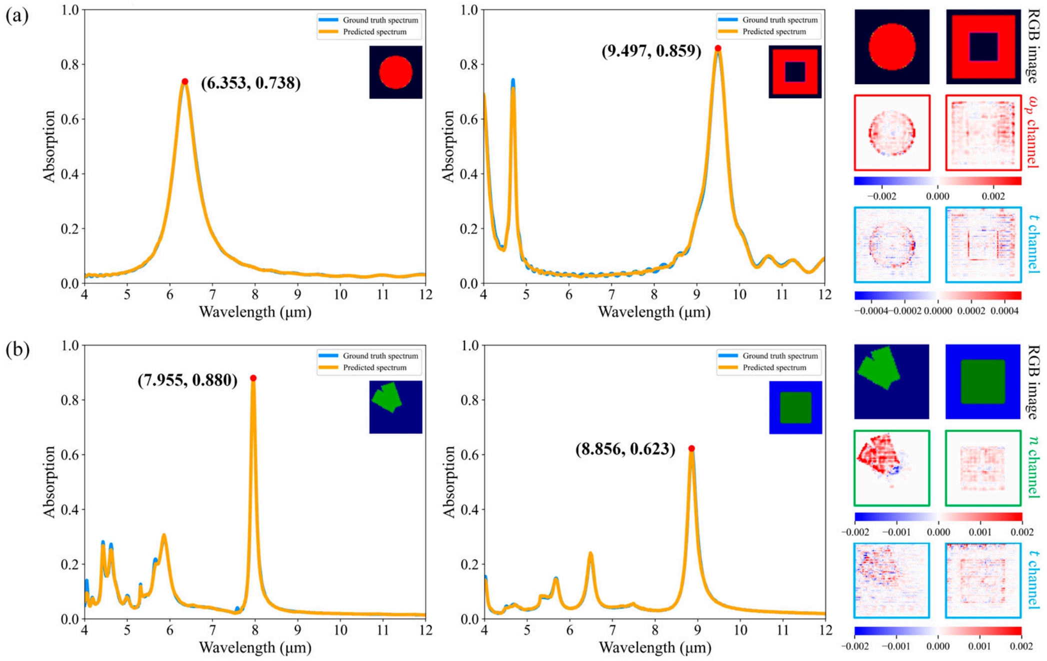

Figure S12. Analysis of the reasons why the model makes predictions. (a) and (b) show the comparison of predicted spectra and ground truth spectra with SHAP values heatmaps at the wave peaks of MIM structures and hybrid dielectric structures, respectively.

This way of visualization can accurately and intuitively tell the designer what is driving

the model's prediction, with more red dots indicating more activation of neurons at the corresponding wavelengths and, conversely, blue dots inhibiting neuron activation. As shown in the figure, the model effectively extracts the required information from the different channels. For the MIM structures, the model mainly focuses on the edge of the resonator shape as well as the material property (plasma frequency) inside the ω_p channel and also focuses on the features outside the resonator (thickness of the dielectric layer) in the t channel. The SHAP values of the pixel points in the t channel are generally smaller, indicating that the model pays more attention to the ω_p channel. For the hybrid dielectric structures, similar to the MIM structures, the model mainly focuses on the resonator shape edges as well as the material properties inside the resonator in the ω_p channel and focuses on the features outside the resonator (thickness of the dielectric resonator) in the t channel. However, the shape outline in the ω_p channel is more blurred, and the number of points falling outside the shape is higher, which suggests that the samples of the mixed-media structure will pay more attention to the thickness features of the dielectric resonator compared to the MIM structure.

To explore which channels contribute more to the prediction, we randomly selected multiple metasurface structures and calculated each channel's absolute average SHAP value separately, as shown in Figure S13.

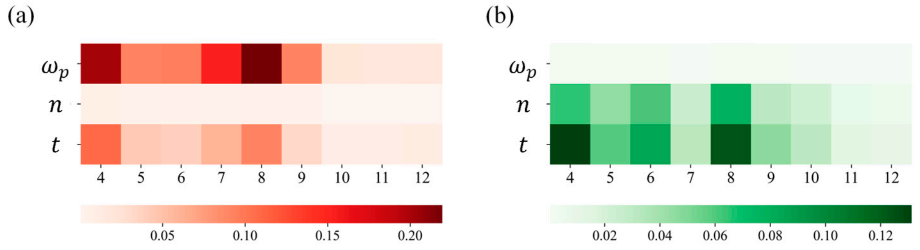

Figure S13. SHAP Global explanations on image channels. (a) and (b) show the absolute average SHAP values at different wavelengths of MIM structure and hybrid dielectric structures, respectively.

For the MIM structures, the average absolute SHAP value of the ω_p channel is larger than that of the t channel, which indicates that the ω_p channel has a more significant influence on the final output predictions, i.e., the model pays more attention to the plasma frequency of the metal material of the resonator compared to the thickness of the dielectric layer. For the hybrid dielectric structures, the absolute average SHAP values of the t channel are larger than that of the n channel, which suggests that the t channel has a more significant influence on the final output predictions, i.e., the model pays more attention to the thickness of the dielectric resonator compared to the actual refractive index of the material of it. The conclusions drawn from the global explanations are consistent with the local explanations and can give a reference for designers to adjust the parameters to design the initial structure.

10. Detail of Ablation study

The architecture of the EP Network without Decoder part is shown in Figure S14. We

removed the Decoder part of the EP Network and directly output the predicted spectrum through a fully connected layer and a sigmoid activation function after the adaptive average pooling layer.

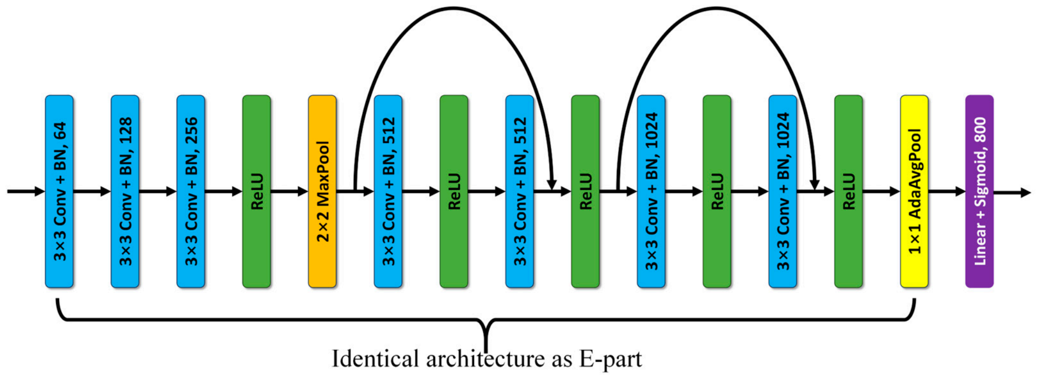

Figure S14. Architecture of the EP Network without Decoder part in Section 3.4.

The architecture of the EP Network without AdaptvgPool and EP Network with multi-channel feature map are shown in Figure S15 and Figure S16, respectively.

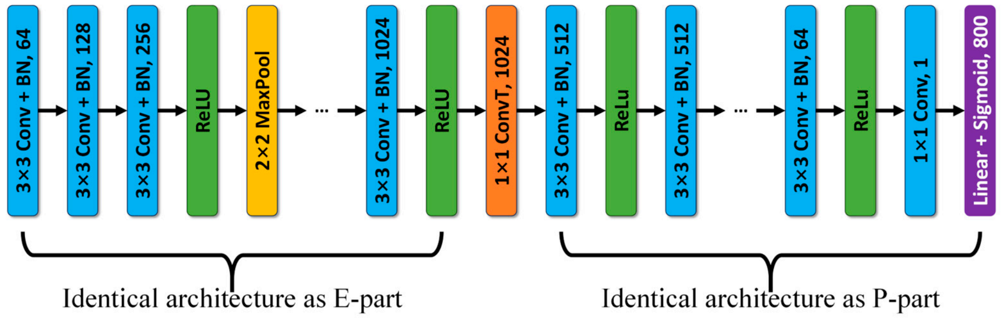

Figure S15. Architecture of the EP Network without adaptive average pool in Section 3.4.

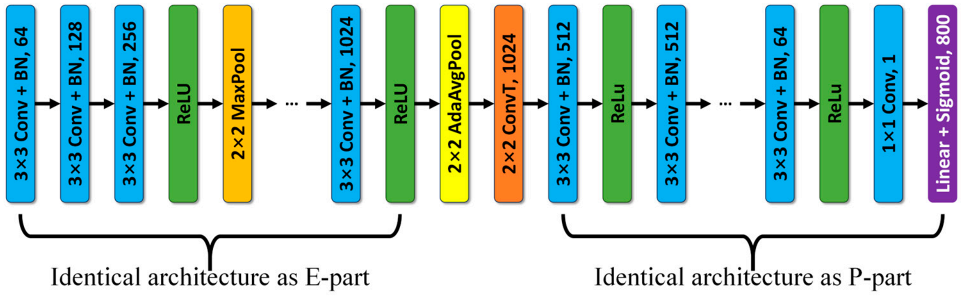

Figure S16. Architecture of the EP Network with multi-channel feature map in Section 3.4.

To investigate the impact of the learning rate for the E-part on the performance of the EP network, we conducted experiments with learning rates ranging from 0 to 0.001, as shown in Table S6. Both SOC loss and MSE loss reach their minimum when the learning rate for the E-

part is set to 0.0006. This indicates that a learning rate of 0.0006 for the E-part provides the optimal balance between SOC and MSE losses in this setup.

Table S6. Test Losses with Varying Learning Rates for E-part.

Learning rate of E-part	Learning rate of P-part	Test loss (SOC)	Test loss (MSE)
0	0.001	0.092	3.896×10^{-4}
0.0001	0.001	0.082	3.053×10^{-4}
0.0002	0.001	0.082	2.980×10^{-4}
0.0003	0.001	0.081	2.976×10^{-4}
0.0004	0.001	0.081	3.002×10^{-4}
0.0005	0.001	0.081	2.923×10^{-4}
0.0006	0.001	0.079	2.843×10^{-4}
0.0007	0.001	0.082	2.917×10^{-4}
0.0008	0.001	0.082	3.061×10^{-4}
0.0009	0.001	0.083	3.281×10^{-4}
0.0010	0.001	0.082	3.053×10^{-4}

11. Exploration of Limitations

To explore the limitations of the model, we conducted a further investigation into the loss distributions. Figure S17 displays the distributions of loss (SOC and MSE) on the test set. Most of the samples on the test set exhibit a prediction loss (SOC) of less than 0.1, with a mean value of 0.079. The mean value of MSE is 2.843×10^{-4}. For MIM structures, the average SOC is 0.057, the average MSE is 1.229×10^{-4}, and for hybrid dielectric structures, the average SOC is 0.128, and the average MSE is 6.230×10^{-4}.

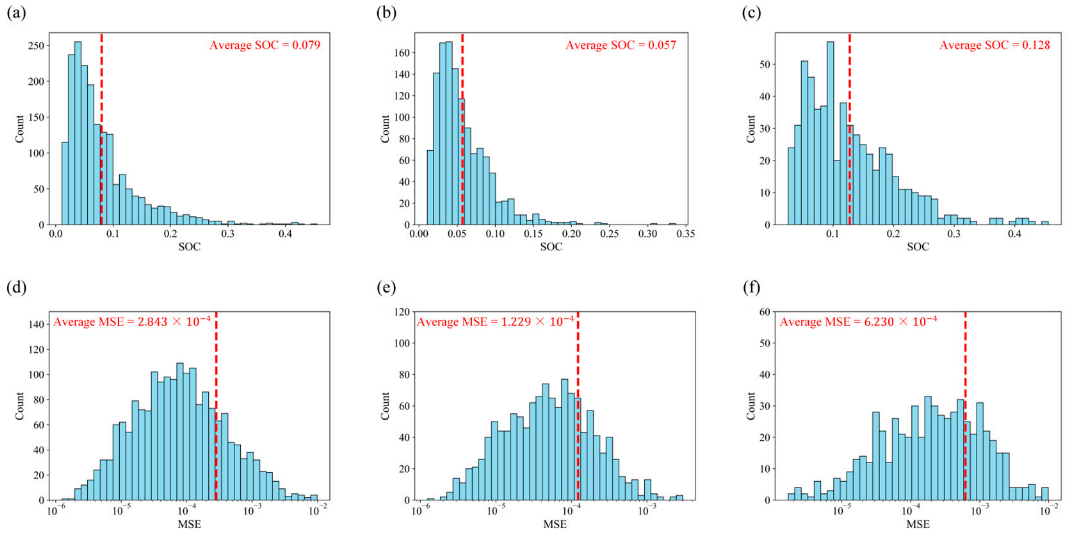

Figure S17. Histogram of loss distribution. (a), (b) and (c) show the SOC distribution for all samples, all MIM structure samples, and all hybrid dielectric structure samples, respectively. (d), (e) and (f) show the MSE distribution for all samples, all MIM structure samples, and all hybrid dielectric structure samples, respectively.

In the test set, we selected the 100 samples with the highest MSE and found that only 11 of them were MIM structures, while the remaining were all hybrid dielectric structures. This indicates that the model's accuracy in predicting the absorption spectra of hybrid dielectric

structures is lower than that for MIM structures. We selected the 10 MIM structures and 10 hybrid dielectric structures with the highest MSE loss, as shown in Figure 18 (a). In the figure, we observe that some yellow or white pixels are present in the images representing the metasurfaces. Each pixel in the images should only contain one color component (red, green, or blue), according to the encoding described in the dataset. Specifically, images representing MIM structures should only have red and blue pixels, while images representing hybrid dielectric structures should only have green and blue pixels. The presence of yellow or white pixels indicates encoding errors in some samples of the dataset. This problem may lead to the model extracting incorrect physical features from the metasurface images, thereby reducing prediction accuracy. Additionally, the absorption spectra of many mixed media structures are more complex, featuring multiple peaks that make accurate fitting by the model more challenging, as shown in Figure S18 (b). Furthermore, the dataset contains fewer samples of hybrid dielectric structures (approximately 32 % of the total), which may also contribute to the model's suboptimal performance in predicting the absorption spectra for this type of metasurface.

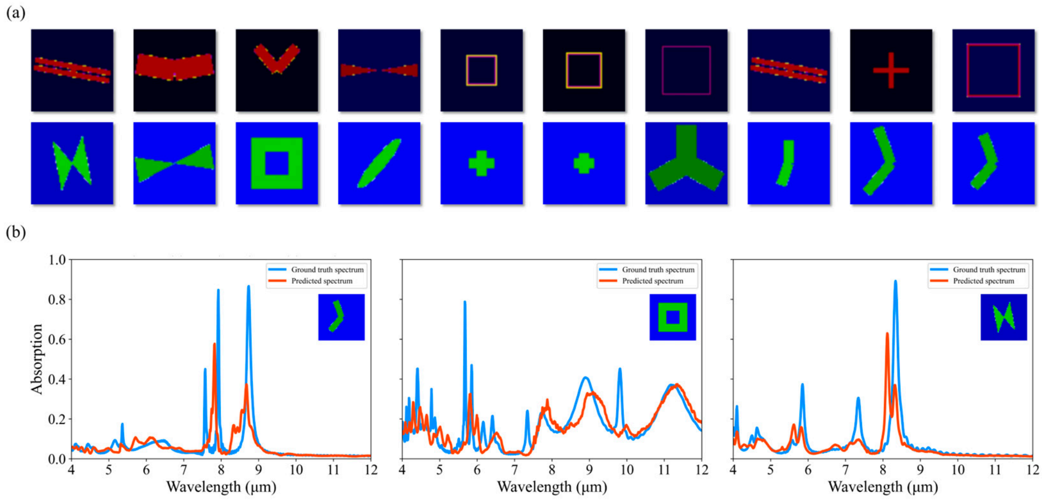

Figure S18. Limitations of model prediction for absorption spectra. (a) The 10 samples with the highest MSE loss for both types of structures. (b) The multi-peak spectra for hybrid dielectric structures.

References

1. Zeng Y, Cao H, Jin X. Anchor-Controlled Generative Adversarial Network for High-Fidelity Electromagnetic and Structurally Diverse Metasurface Design[J]. arXiv preprint arXiv:2408.16231, 2024.
2. Lundberg S M, Lee S I. A unified approach to interpreting model predictions[J]. Advances in neural information processing systems, 2017, 30.
3. Shapley L S. A value for n-person games[J]. 1953. DOI:10.1515/9781400829156-012.
4. Lundberg S M, Nair B, Vavilala M S, et al. Explainable machine-learning predictions for the prevention of hypoxaemia during surgery[J]. Nature biomedical engineering, 2018, 2(10): 749-760. DOI:10.1038/s41551-018-0304-0.
5. Yeung C, Tsai R, Pham B, et al. Global inverse design across multiple photonic structure classes using generative deep learning[J]. Advanced Optical Materials, 2021, 9(20): 2100548. DOI:10.1002/adom.202100548
